# EDISP: a hybrid CNN-ViT framework for robust maize leaf disease detection and classification

**DOI:** 10.3389/fpls.2026.1873194

**Published:** 2026-07-10

**Authors:** Aziz Ullah, Shah Hussain, Qi Hou, Di Fang, Leilei Deng

**Affiliations:** 1 College of Information and Technology, Jilin Agricultural University, Changchun, China; 2 Department of Software Engineering, Bahria University, Islamabad, Pakistan; 3 College of Computer Science and Technology, Changchun University of Science and Technology, Changchun, China

**Keywords:** crop disease classification, deep learning, hybrid CNN-ViT model, intelligent plant protection, maize disease detection, precision agriculture

## Abstract

**Introduction:**

Maize is one of the most important food crops in the world, and foliar diseases can lead to significant yield losses if identification is not performed on time. Experts conducting manual inspections find it less effective and more subjective. Deep learning-based approaches utilizing Convolutional Neural Networks (CNNs) and Vision Transformers (ViTs) have been demonstrated as a viable approach to automate disease diagnosis. Thus, while CNNs fail to capture wider context due to their local feature focus and ViTs need larger datasets and tend to miss finer-grained details. To overcome these limitations, we present EDISP a hybrid framework that connects Convolutional Neural Networks (CNNs) and Vision Transformers (ViTs) for local feature extraction, as well as global contextual learning.

**Methods:**

The EDISP framework brings together the strengths of CNNs and ViTs to overcome their individual weaknesses. It is trained on a dataset that includes both controlled-environment and real-field maize leaf images, which helps it handle different environmental conditions. The data undergoes thorough preprocessing, including normalization, augmentation, and stratified splitting into training, validation, and test sets to support generalization. The CNN focuses on detailed local disease features, while the ViT captures broader contextual information across the maize leaf surfaces.

**Results:**

The proposed EDISP model significantly outperforms standalone CNN and ViT Models in multiple performance metrics, achieving an overall classification accuracy of 99.40%, precision of 99.43%, recall of 99.38%, and an F1-score of 99.40%. Experimental results demonstrate that EDISP excels in identifying maize leaf diseases, including Common Rust, Gray Leaf Spot, Northern Leaf Blight, and Healthy leaves, with minimal false positives and negatives. External validation with an independent dataset further highlights the model’s robustness and ability to generalize to real-world conditions.

**Discussion:**

The EDISP hybrid architecture, integrating CNNs and ViTs, provides a stronger method for accurate, automated maize leaf disease detection. Its robust performance, consistent results on controlled and field datasets shows robustness in diverse environments. However, EDISP’s effectiveness may be limited by image quality, lighting, or disease types not seen in training. These results highlight the promise of hybrid deep learning in precision agriculture and offer a scalable solution for disease detection, supporting farmers without expert diagnostic resources.

## 1 Introduction

Agriculture remains one of the largest contributors to global food security, as the world population is projected to reach 9.7 billion by 2050 FAO et al. (2024). To meet the increased demand for food, crop productivity needs to be increased by about 70%. Maize (*Zea mays* L.) is a major staple crop with significant nutritional and economic value. Maize is native to the Balsas region of southwest Mexico Aravind et al. (2020). Maize is now widely cultivated by many countries, with the leading producers being the USA, followed by India Sahu and Amudha (2022). Maize is one of the most important crops in the world and is heavily affected by foliar diseases, including the maize disease complex, which consists of Common Rust, Gray Leaf Spot, and Northern Leaf Blight. Annual yield losses from these diseases are estimated at 14-15%, with further increases during epidemics Dash et al. (2023).

Traditionally, maize disease diagnosis is performed through manual inspections by experts, a timeconsuming and subjective procedure that may not be feasible for small-scale farmers or farmers in rural settings Yadav et al. (2025). Current deep learning techniques, especially Convolutional Neural Networks (CNNs), have demonstrated encouraging outcomes in automating the identification of plant diseases. CNNs are effective at handling local features like lesion shape, texture, and color Birthal et al. (2025). However, long-range spatial dependencies are difficult to model with CNNs, but are needed when studying diseases that infect larger areas of the leaf. On the contrary, Vision Transformers (ViTs) demonstrated their effectiveness at modeling the global context of an image using a self-attention mechanism Hari et al. (2024); Ferentinos (2018). ViTs are powerful but require large, homogeneous datasets. They also struggle with real-world images that have variable lighting, capture angles, and background noise. Second, most studies do not generalize to the real world Zeng et al. (2023). Hybrid deep learning models have recently gained attention in agricultural image analysis because they combine the strengths of convolutional and transformer-based architectures. For example, a hybrid model integrating convolutional feature extraction with transformer attention mechanisms demonstrated improved classification performance in complex plant phenotyping tasks, outperforming conventional CNN-Only approaches Shawon et al. (2026b) Similarly, another study applied a hybrid framework to crop disease recognition, showing that augmenting convolutional representations with global context through self-attention reduced misclassification under variable field Al Qwaid et al. (2026) These findings highlight the potential of hybrid architectures in precision agriculture. They also motivate the design of our CNN–ViT-Based EDISP Model for maize leaf disease detection.

In this study, we suggest EDISP, a hybrid framework that can effectively combine CNNs and ViTs for local feature extraction and global context learning. To handle environmental variations, we trained the model on a multi-source dataset containing both controlled and field maize images. Experimental results show that EDISP outperforms standalone CNN and ViT models. External testing indicates that the model generalizes well to unseen real-world conditions. The EDISP framework provides a scalable solution for maize disease detection, making it highly relevant for precision agriculture.

### 1.1 Research gap

Although deep learning techniques have shown consistent performance in plant disease identification, they still have several limitations. CNNs are efficient at learning local spatial features but often struggle to capture long-range dependencies across the entire leaf image. By contrast, Vision Transformers (ViTs) capture global contextual information but often require large datasets because they rely on missing high-level local information. Furthermore, real-world agricultural data are difficult to collect, which limits dataset availability and diversity. As a result, many studies rely on single-source datasets collected under controlled conditions. To overcome these challenges in maize disease classification, this work introduces EDISP, a hybrid CNN–ViT framework that jointly learns local disease representations and global contextual information. The CNN branch captures fine-grained lesion patterns, while the ViT branch models long-range dependencies across the leaf image, improving robust maize disease identification.

### 1.2 Main contributions

The following is a summary of this study’s primary contributions:

1. A novel hybrid deep learning framework, EDISP, that combines CNN-based local feature extraction with Vision Transformer-based global contextual learning for maize leaf disease classification.
2. A multi-source dataset that integrates controlled laboratory images and real-field images to improve model robustness and generalization.
3. A comprehensive experimental evaluation comparing CNN, ViT, and the proposed hybrid model, demonstrating significant performance improvements.
4. External validation of the proposed model to assess real-world applicability and generalization capability.
5. A detailed analysis of the complementary strengths of CNN and transformer architectures for agricultural image classification.

## 2 Related work

Advancements in computer vision and deep learning have transformed plant disease diagnosis. These improvements enhance the accuracy and scalability of agricultural surveillance systems. Over the past decade, the diagnosis of plant diseases using computational methods has undergone significant evolution, motivated by the increasing demand for sustainable agricultural output and efficient early disease management. The application of Vision Transformers (ViTs), Convolutional Neural Networks (CNNs), and hybrid architectures for automatic feature extraction, categorization, and recognition of plant leaf diseases Bhola and Kumar (2025); Zhu et al. (2025). Several investigations have also been conducted to identify diseases in cereal crops. Introduced a hybrid approach that uses both deep learning and machine learning for disease identification on rice, corn, and wheat crops. The authors extract features from a pretrained Densenet201 and classify them using an SVM Model Bhola and Kumar (2025). The results demonstrate exceptional performance, reaching an accuracy score of 87.23% with only 20.2 million parameters.

These methods enable accurate identification of diseased areas, facilitate timely crop management and contributing to sustainable agriculture. CNNs have long been the dominant framework for plant disease detection due to their ability to extract local spatial characteristics, such as texture, venation patterns, and leaf margins. Several CNN architectures have been used in numerous researches to improve classification accuracy for maize and other crop diseases. Proposed a VGG16-based maize disease classification Model.

Tariq et al. (2024) using layer-wise relevance propagation, achieving 94.67% accuracy. Similarly, Pushpa (2024) an AlexNet model was employed that achieved 96.76% accuracy on a dataset of maize, rice, and tomato leaves from PlantVillage. Pratama and Pristyanto (2023) demonstrated the superior performance of MobileNet with an 83.37% accuracy and a geometric mean of 0.8298 for maize leaf classification.

Further expanded CNN applications by incorporating multispectral canopy reflectance data for precise maize disease mapping Zhang et al. (2025). Hybridized CNNs have also shown promise, integrating EfficientNetB0 and DenseNet121 Kinyanjui et al. (2025), achieving 98.56% accuracy, surpassing ResNet152 and InceptionV3. Similarly, Bhola and Kumar (2025) developed a hybrid DLML Model combining DenseNet201 for feature extraction and an SVM classifier, reaching accuracies of 99.82% for corn, 98.75% for wheat, and 84.15% for rice. Enhanced CNN’s inception modules with depth-wise convolutions Chen et al. (2022), yielding an average accuracy of 97.89% and specificity of 98.85% on rice disease datasets.

Recent research highlights the effectiveness of Vision Transformers (ViTs) in plant disease recognition, particularly for large-scale, highresolution image datasets. Unlike CNNs, ViTs leverage self-attention mechanisms to capture long-range dependencies and global contextual information within an image Nishankar et al. (2025); Barman et al. (2024). They developed a smartphone-based ViT application to detect tomato leaf diseases, achieving superior accuracy compared to InceptionV3 Sambana et al. (2025). YOLOv7 and YOLOv8 models outperform earlier architectures for detecting fungal, bacterial, and viral infections in crops, while Han et al. (2025) used a Generative Adversarial Network (GAN) to synthesize additional plant disease images, mitigating dataset limitations. Furthermore, Ali et al. (2024) proposed an ensemble framework that integrates multiple DL models to address class imbalance and boost classification accuracy.

Combining CNN and ViT architectures has recently gained attention for balancing local and global feature extraction. Hybrid models such as ViT and CNN Transformer frameworks integrate the spatial feature sensitivity of CNNs with the self-attention mechanism of ViTs, improving classification robustness Banerjee et al. (2025); Alomar et al. (2025). Such models have demonstrated remarkable accuracy in detecting maize leaf diseases, achieving up to 99% on benchmark datasets Pacal and Is¸ık (2025). More recent hybrid deep learning techniques have further explored effective combinations of convolutional and attention-based representations in agricultural and related image analysis tasks. For example, a hybrid architecture integrating convolutional blocks with transformer modules demonstrated enhanced discrimination between similar disease classes in crop images, and showed improved resilience to noise and background variability compared with standalone CNNs or ViTs Shawon et al. (2026a) Similarly, a study using a multi-stage hybrid network with progressive feature aggregation yielded higher accuracy in complex field datasets, highlighting the benefit of staged interactions between local and global features Shawon et al. (2024) Additionally, recent work in precision agriculture applied hybrid deep models for multi-disease recognition under diverse environmental conditions, reporting significant gains in classification performance and robustness Austin et al. (2025) These developments underscore the increasing value of hybrid approaches and provide further context for our choice to combine key strengths of CNNs and ViTs in the proposed EDISP Model.

Overall, the literature indicates a consistent shift toward transformer-based and hybrid deep learning models that address the limitations of traditional CNNs and ML techniques. This evolution underlines the growing importance of integrating global contextual learning, data augmentation, and cross-domain adaptability for high-precision plant disease detection in diverse agroecological environments. A summary of related studies on deep learning models for maize and plant disease detection is presented in **Table 1**.

**Table 1:** Summary of related studies on deep learning models for maize and plant disease detection (2021–2025).

Ref	Year	Methodology/model	Dataset used	Key findings/accuracy
(Xu et al., 2022)	2022	Support Vector Machine (SVM) for maize seed classification	8,080 maize seed images	Classified five types of maize seeds with 96.46% accuracy
(Fraiwan et al., 2022)	2022	CNN-based transfer learning	Maize leaf dataset	Achieved 98.6% accuracy with biasreduced validation
(Chen et al., 2022)	2022	Inception CNN with depth-wise convolutions	PlantVillage and rice disease dataset	99.21% accuracy (PlantVillage), 95.62% (real-world rice dataset)
(Wang et al., 2025)	2025	Systematic review of deep learning methods	Multiple datasets	Identified DL as state-of-the-art but noted interpretability limitations
(Gopalan et al., 2025)	2025	ResNet152 CNN	Maize leaf dataset	Achieved 98.34% accuracy
(Micheni et al., 2023)	2023	ResNet34 CNN	PlantVillage (4 maize diseases)	Mean accuracy 97.6%, F1-score = 0.93
(Yang et al., 2024)	2024	Cross-domain deep learning analysis	Multi-domain datasets	Achieved 63.95% (1-shot) and 80.13% (5-shot)
(Shahid et al., 2024)	2024	Ensemble deep learning framework	Multiple public datasets	Improved accuracy under class imbalance
(Gómez et al., 2023)	2023	Lightweight CNN architectures	PlantVillage and real-field datasets	Achieved 95–99% accuracy depending on architecture
(Ji et al., 2024)	2024	ICS-ResNet CNN	PlantVillage dataset	98.87% accuracy with 69% fewer parameters
(Yang et al., 2023)	2023	CNN + SVM hybrid model	Rice, maize, wheat datasets	95–99% (rice/maize), lower generalization on wheat (80–85%)
(Zheng et al., 2026)	2026	RGB and spectral survey (CNN/ViT/GAN)	PlantVillage, FGVC datasets	Highlights domain shift challenges and effectiveness of DL methods

## 3 Proposed model

**Figure 1:**
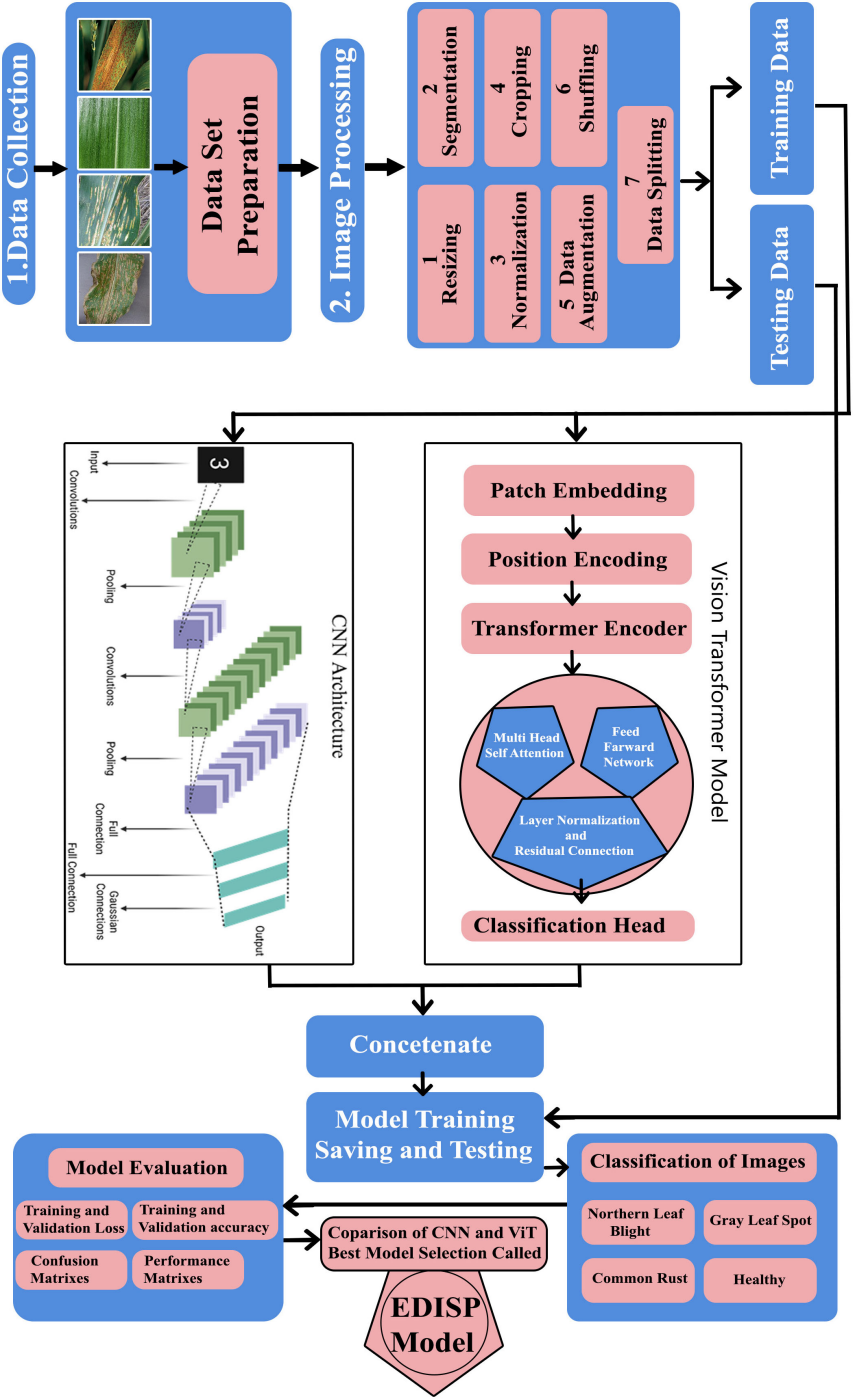
The proposed hybrid EDISP model for plant leaf disease identification and classification.

This section presents the overall workflow of the proposed EDISP (Enhanced Disease Identification and Symptom Prediction) framework for maize leaf disease detection. The entire process includes data collection, preprocessing, model construction, training, evaluation, and deployment, as shown conceptually in **Figure 1**. The proposed EDISP Model for plant leaf disease uses a hybrid architecture that combines CNN and Vit, as shown in **Figure 1**. This architecture identifies both local and global features, improving performance across disease classes. The following detailed process outlines each step of our Model from input preprocessing to final classification. The EDISP framework transforms raw images into actionable agronomic insights through a multi-stage pipeline. At its core, the Model combines a hybrid deep learning backbone integrating Convolutional Neural Networks (CNNs) and Vision Transformers (ViTs) with a temporal-environmental predictive layer. In this study, data are collected from various reliable sources. These images contain a wide range of plant species to cover various diseases. As the objective of this Model is to develop a generalized DL Model, this phase focused on image quality and diverse image sets. The model was trained and tested using two datasets: the Maize or Maize Leaf Disease Detection dataset from Kaggle and the Maize Leaf Disease Detection dataset from Mendeley. The four kinds of maize leaf diseases examined in this work are included in these databases. Following data collection, the images undergo preprocessing to eliminate undesired noise and transformation in accordance with model specifications. The photos are downsized to a consistent 256 × 256-pixel size during preparation. After that, the pictures are normalized in the (0, 1) range. Data pre-processing performs a series of steps on raw images to prepare them for further processing. The objective of this step is to improve image quality and reduce noise and irrelevant information that might hinder the Model’s performance. The following steps are performed in this phase: The photos undergo data augmentation, which includes arbitrary rotation and flipping. In order to remedy the class imbalance, this step increases the number of photos. In order to stop a pattern from reoccurring, the pictures are then shuffled. After that, the pictures are divided into three sets for training, testing, and validation at an 8:1:1 ratio. The photos are prepared to be fed into the model for training after these preprocessing stages. There are two components to the suggested methodology. First, a CNN Model is constructed to detect local features in the pictures. The model design uses convolutional layers, maxpooling layers, dropout layers, and fully connected dense layers to classify input maize leaf images into four different disease categories. The CNN Model is then trained and evaluated using the preprocessed images. The dataset is split into three separate subsets using data splitting: (i) train, (ii) validate, and (iii) test. The training dataset comprises 80% of the total data. Model learns patterns, features, and relationships from training data and minimizes the loss function. 10% of the total data is used for validation, which helps with Model tuning and prevents overfitting. The remaining 10% of the data is used for testing to evaluate the final Model’s performance.

Model Building is the core of the proposed Model. **Figure 1** shows the architecture of the proposed EDISP Model. It can be observed that the input image is processed through parallel CNN and ViT Branches. The CNN Branch extracts local features such as texture and lesion patterns, while the ViT Branch captures global contextual dependencies. The extracted features are then fused to produce the final classification output. The pre-processed image is passed in parallel through two branches: (i) CNN Branch, and (ii) Vit Branch. Thereafter, the outputs from these two branches are merged and passed through the output layer to produce the final result. The goal of this project is to develop a CNNViT hybrid model. The ViT framework is added to capture global contextual associations inside image patches, while the CNN component is kept for local feature extraction. After that, the CNN and ViT outputs are combined and passed through dense layers for classification. The output feature maps from the CNN and ViT are concatenated to generate a single feature vector. The concatenated attributes are processed through fully connected layers in order to find more intricate patterns. The final dense layer uses the classification findings to predict the maize disease category for each leaf image.

The hybrid CNN + ViT Model will next be trained and assessed using the same datasets. The test dataset is used to evaluate both models. The same performance criteria are used to compare the classic CNN and the hybrid model. Lastly, an output comparison between the CNN and hybrid models is shown, emphasizing the hybrid architecture’s improvements in accuracy and overall classification performance. The benefits of the hybrid model for disease classification tasks are emphasized in the final analysis, which summarizes the findings. The requirement to efficiently extract both global structural patterns and local fine-grained information from plant leaf photos motivated the invention of the suggested EDISP Model. This motivates the integration of the CNN and Vit Branches, each contributing distinct representational strengths. CNNs are particularly effective at capturing spatially localized patterns using convolutional filters. In the EDISP Model, we use six convolutional layers with progressively increasing filter depths and ReLU activation. Max pooling is applied in alternating layers to down sample and retain dominant features. This branch is crucial for detecting localized lesions and edge-level variations, which are typically present in the early stages of the disease. The EDISP Model effectively integrates the localized feature-extraction capability of CNNs with the global-context learning power of transformers, achieving higher accuracy and stronger generalization across maize crop datasets while maintaining computational efficiency suitable for real-world deployment.

### 3.1 Implementation

This section outlines the datasets used to train and evaluate the EDISP Model, including data collection, preprocessing, and augmentation procedures. Given that the model’s success depends on a large, diverse dataset, these datasets were compiled to capture the most relevant environmental conditions for classifying maize leaf diseases.

#### 3.1.1 Experimental setup and system configuration

The proposed EDISP Model was implemented using TensorFlow and Keras frameworks on a GPUenabled system. The experimental setup consisted of a DELL laptop running Windows 11 Pro, Version 25H2 (OS Build 26200.8457) with AMD Ryzen 7 5800H processor, 16 GB RAM, and NVIDIA RTX 3060 Laptop GPU (6 GB). The model contains 8.6 million trainable parameters and processes a 256 × 256 maize leaf image in 28 ms on GPU and 0.18 s on CPU, making it suitable for real-time and edge deployment. Training was performed using the RAdam optimizer, with a batch size of 64, dropout of 0.5, and sparse categorical cross-entropy as the loss function.

#### 3.1.2 Input dataset/dataset details

**Figure 2:**
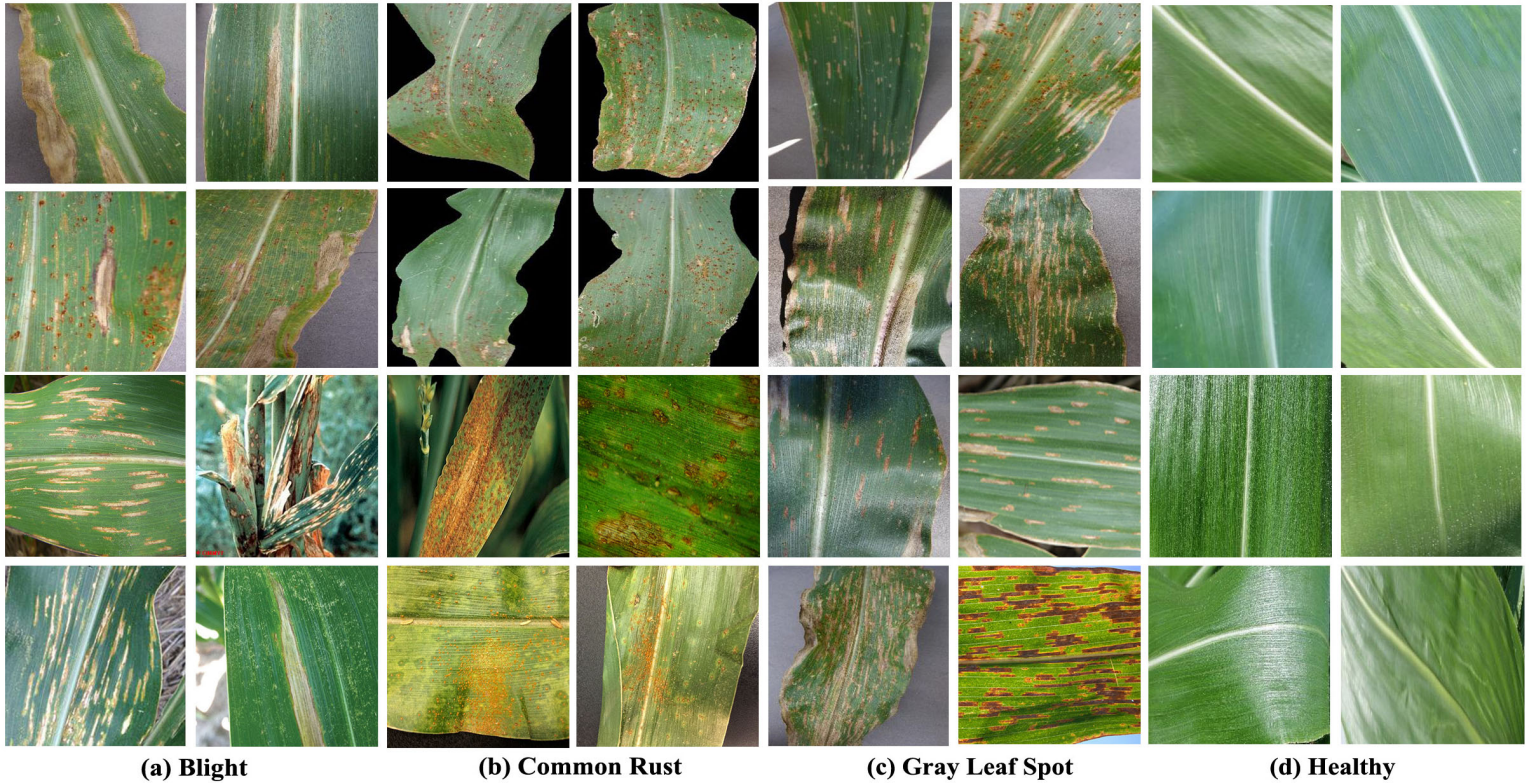
Sample images: **(a)** NLB, **(b)** CR, **(c)** GLS, and **(d)** healthy class.

This study uses two maize leaf disease datasets: the Kaggle Maize Leaf Disease Dataset and the Mendeley Maize Leaf Disease Dataset. The Kaggle dataset, sourced from the PlantVillage repository, contains images collected under controlled conditions, while the Mendeley dataset includes real-field images from Ghana, capturing natural variations in lighting and background. Both datasets contain four disease categories: Common Rust, Gray Leaf Spot (GLS), Northern Leaf Blight (NLB), and Healthy. The combined dataset contains 8,040 images. The Kaggle dataset provides cleaner, higher-quality images, while the Mendeley dataset introduces variability, making the model more robust to real-world conditions. These images were preprocessed to a uniform size of 256 × 256 pixels, normalized, and augmented with transformations like flipping, rotation, and zooming. Stratified splitting was applied to ensure balanced representation across the training, validation, and testing sets. The Kaggle dataset comprises 4,188 images, with 80% used for training, 10% for validation, and 10% for testing. The Mendeley dataset contributes 3,852 images. Both datasets undergo strict quality control, with agricultural experts verifying disease labels and excluding lowquality samples. **Figure 2** shows some photos from the collection.

#### 3.1.3 Mendeley maize leaf disease dataset

The Mendeley dataset contains 3,852 images, specifically selected from the original dataset of 39 plant diseases. The images were captured under field conditions in Ghana, incorporating environmental variability. The dataset is divided into four classes: Healthy, NLB, GLS, and CR. Each image was labeled by experts and reviewed for quality, with only those exhibiting clear disease symptoms retained. This dataset helps evaluate model performance under more complex, realworld conditions.

#### 3.1.4 CD&S external benchmark dataset

For external validation, the CD&S dataset includes 1,597 raw field images collected at Purdue University. These images cover NLB, GLS, and Northern Leaf Spot (NLS). Unlike the Kaggle and Mendeley datasets, this dataset was used exclusively for testing, providing an objective evaluation of model generalization to unseen data with variations in lighting and background.

#### 3.1.5 Overall dataset

**Figure 3:**
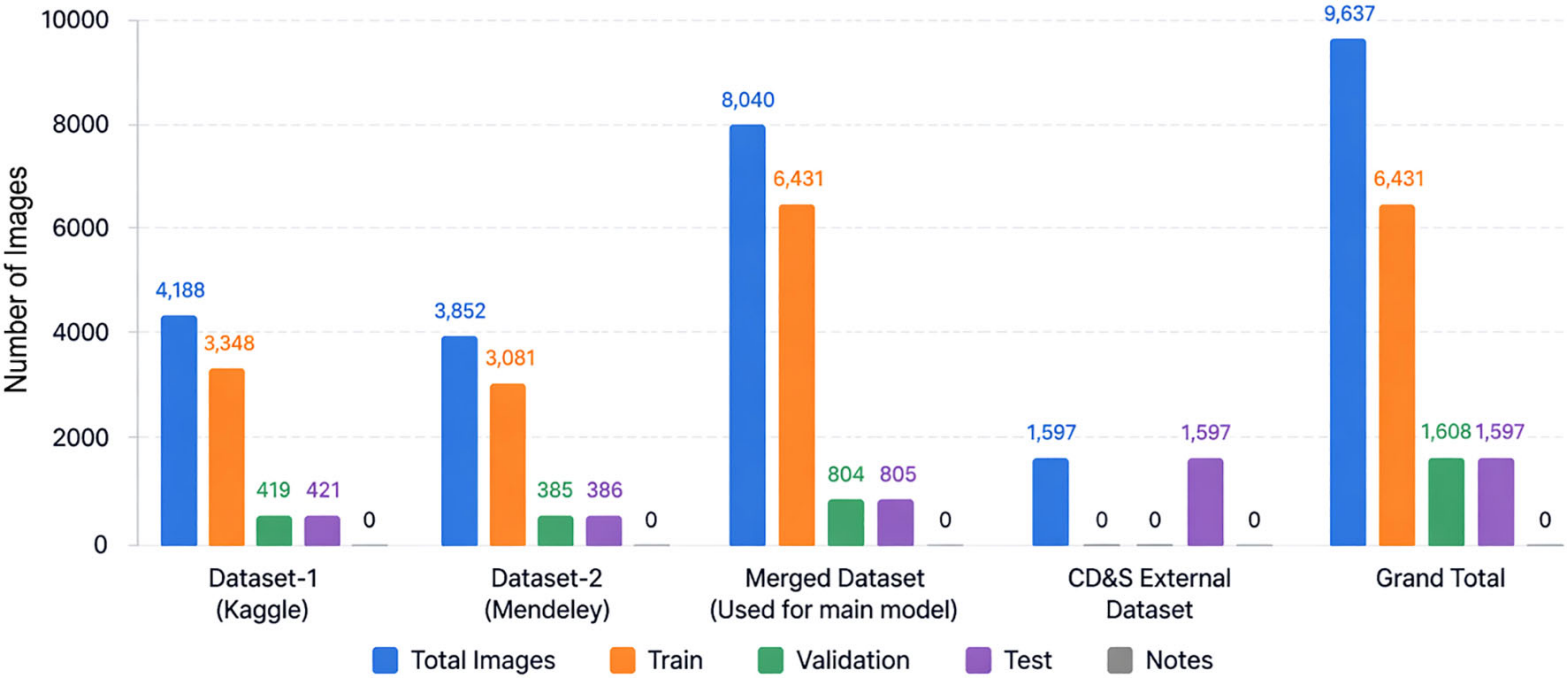
Represents the graphical representation of the overall dataset summary.

Three supplementary datasets were used to provide a thorough and objective assessment of the proposed classification system, as shown in **Table 2** and **Figure 3**. The final dataset consists of 9,637 images across three sources: Kaggle (4,188 images), Mendeley (3,852 images), and CD&S (1,597 images). The training dataset comprises 6,431 images, with 804 for validation and 805 for testing, split using an 80:10:10 ratio. This diverse dataset ensures the model’s robustness to both controlled and real-world conditions, and its external validation confirms its ability to generalize.

**Table 2:** Overall dataset summary.

Dataset	Total Images	Train	Validation	Test	Notes
Dataset-1 (Kaggle)	4188	3348	419	421	Controlled environment images
Dataset-2 (Mendeley)	3852	3081	385	386	Real-field Ghana images
Merged Dataset (Used for main model)	8040	6431	804	805	Used for CNN and Hybrid CNN-ViT
CD&S External Dataset	1597	–	–	1597	External benchmark only
Grand Total	9637	–	–	–	–

This dataset provided an objective assessment of the Model’s resilience to previously unseen samples, as it was not used during training or validation. Thus, a thorough, high-fidelity evaluation of the suggested approach was ensured by integrating multiple data sources and imaging settings.

#### 3.1.6 Data preprocessing and augmentation

To maintain consistency across datasets and reduce domain bias, a unified preprocessing pipeline was applied to all images. This pipeline includes resizing, segmentation, normalization, cropping, and data augmentation to ensure that the model learns relevant disease-related features while maintaining robustness across varying environmental conditions **Figure 2** illustrates the preprocessing pipeline used in this study. It can be observed that the input images undergo resizing, normalization, and augmentation before being fed into the model. These steps improve data consistency and enhance model generalization.

#### 3.1.7 Resizing

The images were resized to 256 × 256 pixels to ensure uniformity across the dataset and balance the need to retain fine-grained disease features with efficient computation during training. Linear interpolation was used for resizing, defined mathematically as:

(1) $$I\left(x,y\right)=\sum_{i=0}^{1}\sum_{j=0}^{1}w_{ij}.I\left(x_{i},y_{j}\right)$$

#### 3.1.8 Segmentation

Segmentation isolates the region of interest from the background, allowing the Model to focus on the leaf area where disease is apparent. The thresholdbased segmentation method is used to isolate the leaf region from the background. Segmented Image = 1 if I (x, y) ¿ T 0 otherwise I (x, y): Intensity of pixel at coordinates (x, y). T: threshold value used for segmentation. The threshold value t is set to 128, which corresponds to the midpoint of the 8-bit pixel intensity range [0,255]. This value is empirically determined to effectively separate the leaf region from the darker background in most images. it balances segmentation accuracy and computational efficiency across the dataset.

#### 3.1.9 Normalization

Normalization is applied to pixel values to ensure a consistent distribution of data across the dataset. This step ensures that each pixel contributes equally to the Model training process, accelerating convergence and reducing gradient issues.

(2) $$X_{\text{norm}}=\frac{X-\mu }{\sigma }$$

X: original pixel value, *µ*: Mean of the pixel values in the dataset, *σ*: Standard deviation of the pixel values in the dataset. The input images are normalized using the standard mean and standard deviation values *µ* = [0.4850, 0.4560, 0.4060] and *σ* = [0.2290, 0.2240, 0.2250]. These values are chosen to align with the normalization expectations of the pretrained CNN and Vit components used in the EDISP architecture. This alignment facilitates stable fine-tuning and improves convergence.

#### 3.1.10 Cropping

Cropping removes unnecessary background information from the image, thereby improving the model’s performance by focusing only on the disease-affected regions.

#### 3.1.11 Data augmentation

A sophisticated data augmentation pipeline was employed to increase the diversity of the training dataset and enhance the model’s generalization capabilities. The augmentation techniques applied include:

Geometric augmentations: Random rotations (± 30^◦^), horizontal and vertical flips, and zooming (± 10%).

Photometric augmentations: Adjustments to brightness and contrast (scaling factors between 0.8 and 1.2).

Advanced augmentations: Techniques like Random Erasing, Color Jitter, and Gaussian Blur were applied to simulate occlusions, shadowing, and field conditions.

CutMix and MixUp: These methods were used to combine multiple samples, thereby increasing the model’s robustness by improving invariance to complex textures and background noise. By simulating realistic field variability, these augmentations allow the model to learn stronger, more discriminative features, making it more resilient to variations in real-world agricultural environments. These augmentations were applied randomly during training. Each image could undergo one or more augmentation operations in random order, increasing the variability of the training dataset and improving the model’s generalization to unseen field images.

#### 3.1.12 Shuffling of images

To reduce any possible class-ordering bias, the images were randomly shuffled before training. This randomization was performed using the FisherYates shuffling algorithm, which ensures a truly randomized sequence by swapping image positions using randomly generated indices. This procedure increases sample diversity during training, enhances model generalization, and reduces the risk of overfitting.

#### 3.1.13 Data splitting

The dataset was split into three subsets 80% for training, 10% for validation, and 10% for testing, to ensure balanced representation of each disease class. The dataset classes are relatively balanced, and the augmentation and shuffling applied prior to splitting ensured sufficient diversity in each class. Therefore, no additional class-balancing methods were required. Stratified sampling was used to preserve the distribution of disease categories across these subsets. The training set was used to learn patterns and minimize the loss function, the validation set guided hyperparameter tuning and model optimization, and the test set was reserved for final evaluation. This data splitting method ensures reliable model evaluation and accurate performance assessment.

#### 3.1.14 CNN branch

Image *x* is passed through a series of 6 convolutional layers. The input image size considered for model processing was 256 × 256 × 3, where 256 × 256 represents the spatial resolution and 3 represents the RGB color channels. In the CNN branch, all convolutional layers used 3 × 3 kernels with stride 1 and padding to preserve spatial information during feature extraction. These kernels were selected to capture local disease features, including lesion edges, texture variations, and venation patterns, while keeping the model computationally efficient. Each convolutional layer applies a set of filter weights *W_i_* to the input feature maps *F_i_*_−1_ to produce the output feature maps *F_i_*. The operation of each convolutional layer is defined as follows:

(3) $$F_{i}=\sigma \left(W_{i}\times F_{i-1}+b_{i}\right)$$

Where *σ* denotes the activation function, typically ReLU (Rectified Linear Unit). After every two convolutional layers, a max pooling layer is applied to reduce the spatial dimensions. The max pooling operation is defined by:

(4) $$F_{\text{pool}}=\max\left(F_{\text{region}}\right)$$

A series of convolutional and pooling layers captures local features at multiple levels of abstraction.

#### 3.1.15 ViT model

By representing an image as a series of patches and using a self-attention mechanism to learn its fundamental structure, the Vision Transformer (ViT) departs from conventional convolution-based feature extraction. ViT uses global associations across the entire image rather than local receptive fields, enabling it to detect subtle, irregular, or dispersed disease patterns across multiple leaf regions more effectively.

Patch Generation and Linear Embedding: Given an input image $I\in {\mathbb{R}}^{H\times W\times C}$, the image is divided into fixed-size, non-overlapping patches of size *P* × *P*. The total number of patches is:

(5) $$N=\frac{H\times W}{P^{2}}$$

Each patch is flattened into a vector $x_{i}\in {\mathbb{R}}^{P^{2}C}$ and projected into a *D*-dimensional embedding through a learnable linear mapping:

(6) $$Z_{i}=x_{i}W_{e}+b_{e}$$

Addition of Positional Encoding: Transformers lack an inherent notion of spatial order because selfattention treats all elements as a set rather than a sequence. To preserve the spatial arrangement of the patches, a learnable positional vector is added:

(7) $$Z_{i}=Z_{i}+E_{\text{pos},i}$$

Multi-Head Self-Attention Mechanism: Selfattention is computed as:

(8) $$\text{Attention}\left(Q,K,V\right)=\text{softmax}\left(\frac{QK^{T}}{\sqrt{d_{k}}}\right)V$$

where *Q*, *K*, and *V* denote the query, key, and value matrices, respectively, and *d_k_*is the dimensionality of the keys. Multi head attention allows the Model to jointly address information from different representation subspaces at different positions.

Where:

(9) $$Q=ZW_{Q},\;K=ZW_{K},\;V=ZW_{V}$$

Multiple attention heads are concatenated:

(10) $$\text{MHSA}\left(Z\right)=\text{Concat}\left({\text{head}}_{1},\ldots ,{\text{head}}_{h}\right)W_{O}$$

A residual connection is applied:

(11) $$Z^{\prime }=Z+\text{MHSA}\left(\text{LayerNorm}\left(Z\right)\right)$$

Feed-Forward Network (FFN):

(12) $$\text{FFN}\left(x\right)=W_{2}\;\text{GELU}\left(W_{1}x+b_{1}\right)+b_{2}$$

Second residual connection:

(13) $$Z^{\prime \prime }=Z^{\prime }+\text{FFN}\left(\text{LayerNorm}\left(Z^{\prime }\right)\right)$$

Classification Token and Output Layer: A CLS token is prepended:

(14) $$Z_{0}=\left[Z_{\text{CLS}},Z_{1},Z_{2},\ldots ,Z_{N}\right]$$

The CLS output:

(15) $$z_{\text{CLS}}^{\left(L\right)}$$

Classification is performed as:

(16) $$y=\text{SoftMax}\left(W_{\text{CLS}}z_{\text{CLS}}^{\left(L\right)}\right)$$

The SoftMax function is defined as:

(17) $$P\left(y_{i}\right)=\frac{e^{z_{i}}}{\sum_{j}e^{z_{j}}}$$

#### 3.1.16 EDISP hybrid model

The EDISP Hybrid Model combines the global, long-range dependency modeling of the ViT Branch with the CNN Branch’s localized feature extraction capabilities. The EDISP architecture creates a more robust and discriminative feature space than either Model by merging these complementary feature streams into a single representation. This fusion strategy is preferred over other hybrid architectures or attention-only methods because it simultaneously leverages complementary strengths while mitigating individual limitations. CNNs efficiently extract fine-grained local features such as lesion edges, texture variations, and venation distortions, which are crucial for early-stage disease detection. ViTs, on the other hand, capture global contextual dependencies across the leaf surface, modeling relationships between spatially distant regions and reducing misclassification of visually similar diseases. Unlike sequential or purely attention-based hybrids, our parallel concatenation ensures that both local and global features are preserved independently before fusion, avoiding dilution of either representation. This design enhances discriminative capacity, reduces overfitting to local noise, and improves robustness across heterogeneous field datasets, providing a balanced representation that consistently outperforms standalone or sequential hybrid architectures. Convolutional filters continue to extract finegrained lesion textures, while the hybrid fusion simultaneously incorporates transformer-based contextual reasoning across the entire leaf surface. In the dual-branch architecture, the CNN Branch produces an output feature map.

The proposed EDISP Model uses a dual-branch hybrid architecture that combines CNN and Vision Transformer features for maize leaf disease classification. After preprocessing, each input image is passed in parallel through the CNN and ViT branches. The CNN branch extracts local disease features such as lesion texture, edges, color variations, and venation patterns, which are important for identifying fine-grained symptoms. In contrast, the ViT branch divides the image into patches and uses self-attention to capture global contextual relationships across the whole leaf surface. The features from both branches are then fused through concatenation. The CNN branch provides a 512-dimensional local feature vector, while the ViT branch provides a 768-dimensional global feature vector. These are combined into a 1280-dimensional hybrid feature representation, which is passed through fully connected layers with ReLU activation and dropout regularization. Finally, a Softmax layer classifies the image into one of four categories: Healthy, Gray Leaf Spot, Northern Leaf Blight, or Common Rust. This hybrid design allows EDISP to use both fine local disease patterns and broader leaf-level context, improving classification performance and reducing confusion between visually similar maize leaf diseases.

**Figure 4:**
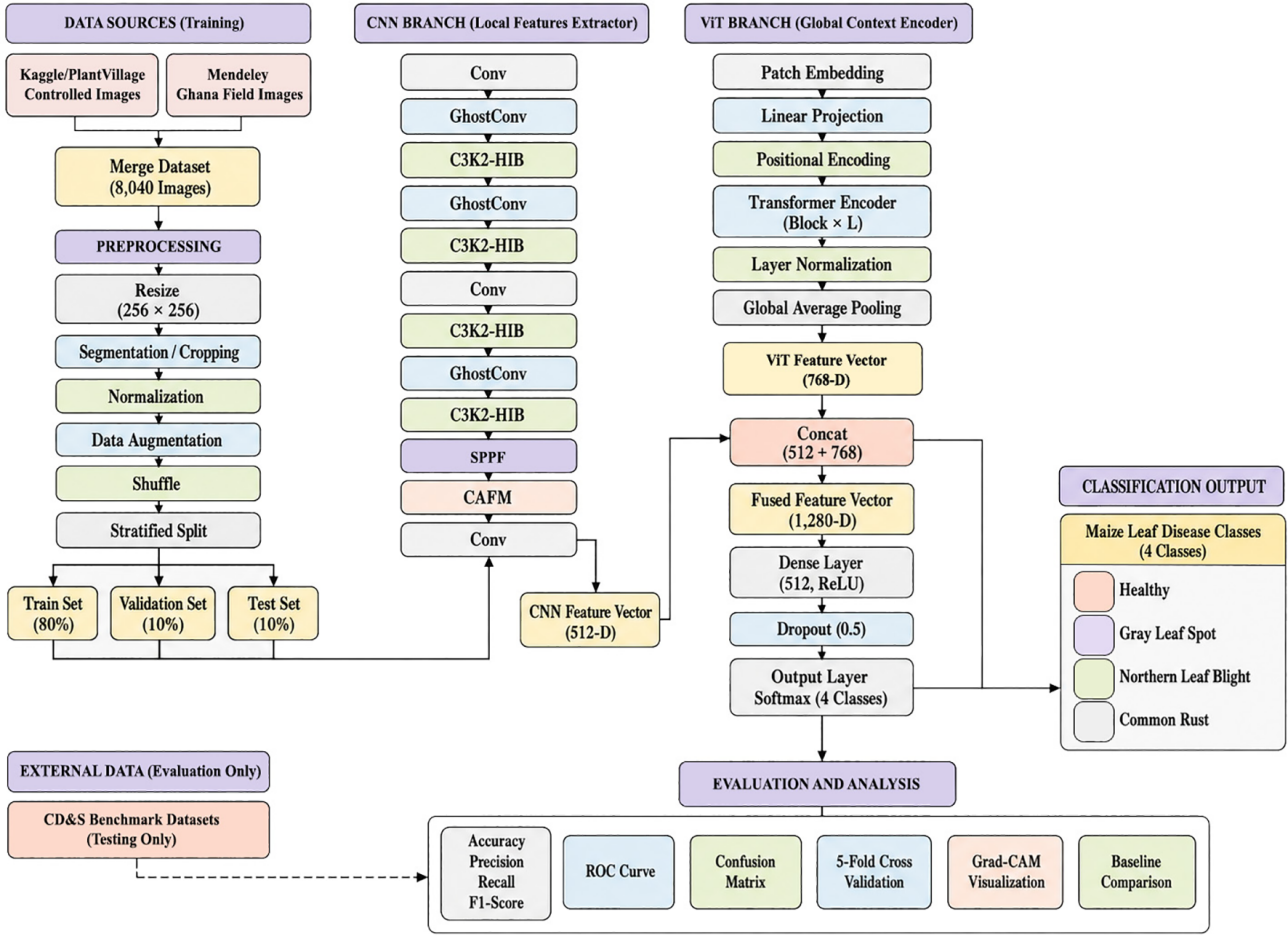
Simplified block-level architecture of the proposed EDISP model, showing CNN and ViT branches, feature fusion, and evaluation pipeline.

The block diagram in **Figure 4** provides a clear overview of the EDISP model architecture, showing the separate CNN and ViT branches, feature fusion, and classification output. This complements the detailed workflow diagram in **Figure 1** and improves the clarity and comprehensibility of the proposed model.

(18) $$F^{\text{CNN}}\in {\mathbb{R}}^{h_{1}\times w_{1}\times d_{1}}$$

Capturing local lesion features with *h*_1_*,w*_1_ denote the spatial dimensions after convolutions and pooling, and *d*_1_ represents the final number of channels.

While the ViT Branch outputs a global feature embedding.

(19) $$F^{\text{ViT}}\in {\mathbb{R}}^{768}$$

which corresponds to the final CLS token representation carrying global contextual information obtained through multi-head self-attention. Before fusion, the CNN feature map is flattened and projected into a 512-dimensional vector to match the representation scale of the transformer output:

(20) $${\hat{F}}^{\text{CNN}}=\text{Flatten}\left(F^{\text{CNN}}\right)$$

(21) $$Z^{\text{CNN}}=W_{c}F^{\text{CNN}}+b_{c}$$

where *W_c_*and *b_c_*denote the learnable linear projection parameters.

The hybrid integration is performed through the concatenation of the projected CNN features and ViT Embeddings, forming a fused vector $F_{\text{Hybrid}}\in {\mathbb{R}}^{1280}$:

(22) $$F^{\text{Hybrid}}=\text{Concat}\left(Z^{\text{CNN}},F^{\text{ViT}}\right)$$

Both the global associations between disease patches throughout the leaf surface (from ViT) and the local lesion margins, edges, and venation abnormalities (from CNN) are encoded in this fused vector. This hybrid feature vector is then processed through two fully connected dense layers: the first with 512 neurons and the second with 256 neurons, using ReLU activation functions and a dropout rate of 0.5 to prevent overfitting.The total number of trainable parameters in the hybrid EDISP Model is approximately 8.6 million, including convolutional, transformer, and dense layer components. These settings were selected to balance model capacity, computational efficiency, and high classification performance across all maize leaf disease classes. A fully connected feed-forward network (FFN) then processes the hybrid features for final classification.

(23) $$H_{1}=\sigma \left(W_{1}F^{\text{Hybrid}}+b_{1}\right)$$

(24) $$H_{2}=\sigma \left(W_{2}H_{1}+b_{2}\right)$$

Here, *σ* is the ReLU activation applied to improve non-linearity and enhance feature discrimination.

Classification Layer: Finally, the output logits for the 4 maize disease classes are produced using:

(25) $$y=\text{Softmax}\left(W_{0}H_{2}+b_{0}\right)$$

This hybrid pipeline enables EDISP to exploit two distinct yet complementary representational spaces, thereby improving the separability between visually similar maize disease categories, such as GLS and NLB. The EDISP Hybrid Model’s fusion technique offers several key benefits that significantly enhance its ability to classify diseases. The hybrid architecture creates a highly discriminative and thorough feature representation by combining the CNN Branch, which is excellent at capturing fine-grained and spatially localized features like lesion edges, venation distortions, and texture variations, with the ViT Branch, which successfully models long-range dependencies and global contextual relationships across the entire leaf surface. The robustness of the Kaggle, Mendeley, and CD&S datasets against domain fluctuations, including lighting variations, background noise, and natural field inconsistencies, is enhanced by this synergistic integration. Additionally, by leveraging complementary information from both local and global feature spaces, the fusion yields more precise decision boundaries, reducing misclassification across visually similar illness categories.

Convolution-driven spatial learning and transformerdriven contextual reasoning work together to improve maize leaf disease detection in the EDISP Hybrid Model, a unified architecture. The Model’s better performance (99.40%) compared to the standalone CNN (97.50%) and standalone ViT (97.90%) is clearly attributed to this synergy, highlighting the benefits of hybrid deep learning for agricultural image processing. While the reported metrics demonstrate excellent performance, we acknowledge potential risks of overfitting and dataset-related biases. The notable accuracy could partly reflect the relatively homogeneous distribution of features within the training and validation sets, so we employed strict separation between training, validation, and external test datasets to mitigate leakage. Additionally, we examined class-wise performance to ensure the model does not favor a particular disease class: precision, recall, and F1-score for all four classes remained balanced, indicating consistent discriminative capability across disease types. Nevertheless, small differences between the ViT and hybrid model highlight that hybridization primarily enhances robustness to subtle local-global feature interactions rather than providing large incremental gains in overall accuracy. Future work could incorporate larger multi-site datasets and cross-validation to further validate generalizability under diverse field conditions.

#### 3.1.17 Performance metrics

Four metrics were used to assess EDISP’s performance: true positive, false positive (FP), true negative (TN), and false negative (FN) Hussain et al. (2023). **Table 3** describes these factors. Based on the parameters, the following performance metrics are calculated: Accuracy measures the percentage of correctly classified instances (infected and healthy leaves) among all instances.

**Table 3:** Representing the evaluation parameters.

Evaluation parameter	Predicted disease	Predicted healthy
Actual Disease	TP (True Positive)	FN (False Negative)
Actual Healthy	FP (False Positive)	TN (True Negative)

(26) $$\text{Accuracy}=\frac{TP+TN}{TP+TN+FP+FN}$$

Precision measures the accuracy of positive predictions (diseased leaves), guaranteeing that high precision suggests a low rate of misdiagnosing healthy leaves as diseased.

(27) $$\text{Precision}=\frac{TP}{TP+FN}$$

Recall measures the Model’s ability to accurately identify all infected leaves, ensuring that high recall minimizes the number of missing unhealthy leaves (false negatives).

(28) $$\text{Recall}=\frac{TP}{TP+FN}$$

F1-score is the harmonic mean of precision and recall, offering a statistic that balances both features. it is crucial in the diagnosis of leaf disease, ensuring accurate identification of infected leaves and minimal misclassification of healthy leaves.

(29) $$\text{F}1\;-\;\text{Score}=\frac{2\times \left(Precision\times Recall\right)}{Precision+Recall}$$

To ensure accurate and dependable automated diagnostic systems, the metrics mentioned above are crucial for assessing the effectiveness of the EDISP intended for leaf disease detection.

## 4 Result and discussion

This section presents the experimental findings of the classification and detection Model for maize disease Diagnosis. The base CNN and the suggested hybrid CNN-ViT Model for classifying maize leaf diseases are thoroughly evaluated in this section. The analysis compares performance with baseline models, conducts ablation tests on architectural configurations, and evaluates the hyperparameter selection and optimization technique.

### 4.1 Optimization strategy and hyperparameter impact analysis

The TensorFlow and Keras frameworks were used to create the suggested Hybrid CNN-ViT Model in a GPU-enabled environment to manage the computational complexity of the Model and speed up training. After experimental adjustment, a batch size of 64 was selected to provide balanced memory utilization and effective convergence. A dropout rate of 0.5 was used to reduce overfitting and enhance Model generalization by randomly turning off neurons during training. To facilitate smoother, more reliable convergence in hybrid architectures, training used the RAdam optimizer, a corrected version of Adam that stabilizes adaptive learning rates during the first training phase. Based on prior research and practical validation, the initial learning rate was set to 1 × 10^−4^. To assess the impact of key parameters, learning rates (1 × 10^−4^, 5 × 10^−4^, 1 × 10^−3^), batch sizes (16, 32, 64), and dropout values (0.3, 0.5), a manual grid search was conducted. Adam, RMSProp, and RAdam were the optimizers examined; RAdam consistently produced better validation accuracy and faster convergence. Since the sparse categorical crossentropy loss function works well for multi-class classification with integer-encoded labels, it was used. The Model demonstrated tolerance for minor hyperparameter tweaks, maintaining consistent performance despite small variations in the learning rate (± 1 × 10^−4^). To improve clarity, the main hyperparameters used for training the proposed EDISP model are summarized in **Table 4**.

**Table 4:** Hyperparameter settings for model training.

Hyperparameter	Selected value/setting	Tested values/details
Input image size	256 × 256 × 3	RGB image input
Optimizer	RAdam	Adam, RMSProp, RAdam tested
Initial learning rate	1 × 10−4	1 × 10−4, 5 × 10−4, 1 × 10−3 tested
Batch size	64	16, 32, 64 tested
Dropout rate	0.5	0.3, 0.5 tested
Loss function	Sparse categorical cross-entropy	Used for four-class classification
Activation function	ReLU	Used in convolutional and dense layers
Output activation	Softmax	Four-class classification
Training epochs	40	Same training setting used for model comparison
Data split	80:10:10	Training, validation, and testing

### 4.2 Deep learning models

**Figure 5:**
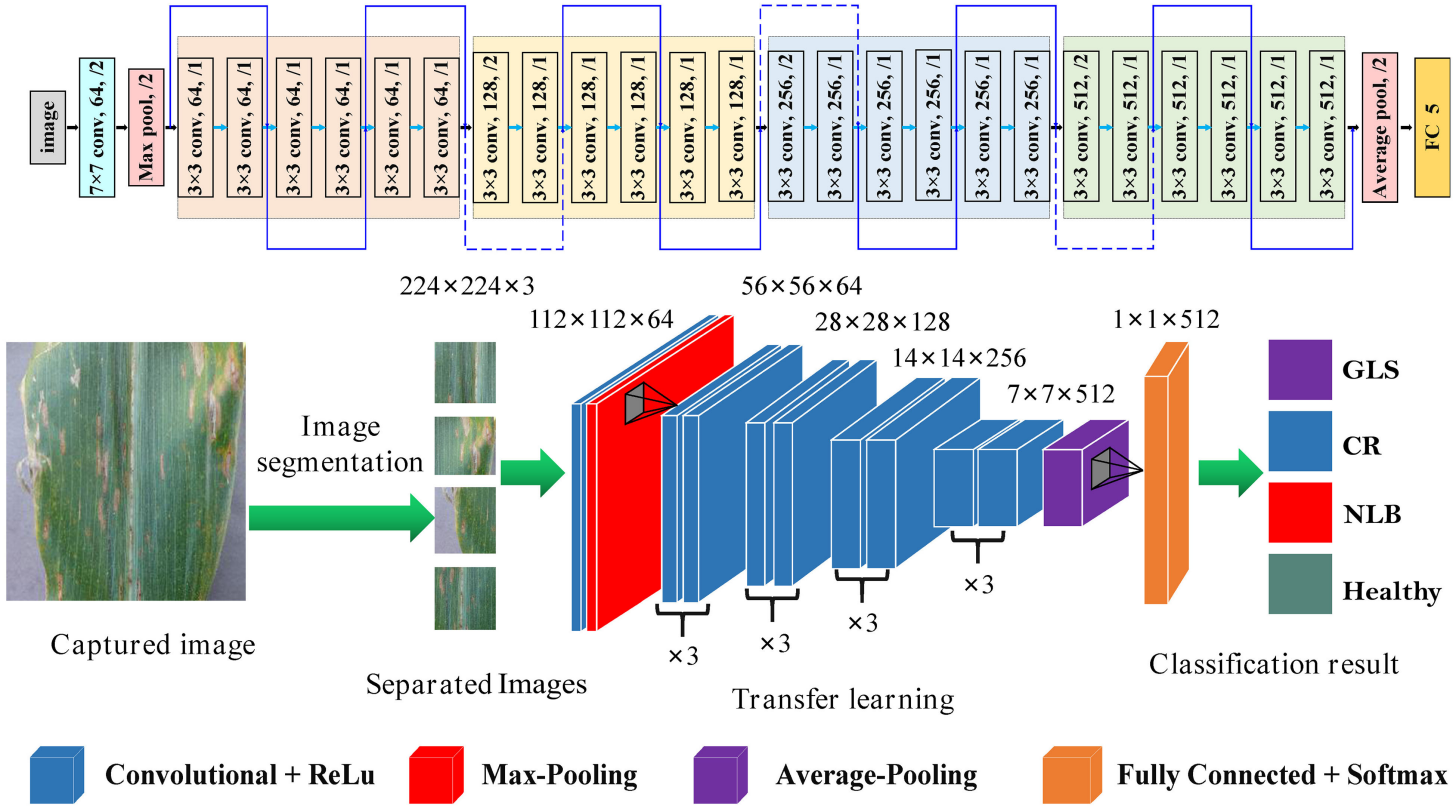
Represent the CNN model architecture.

When classifying maize leaf diseases, the proposed CNN Model demonstrated moderate performance compared to advanced deep learning approaches. The Model achieved training, test, and validation accuracies of 63%, 60%, and 60%, respectively. Additionally, the Model achieved an F1-score of 61%, a precision of 61%, and a recall of 60%, indicating relatively balanced performance between sensitivity and precision. Its overall accuracy of 60.05% reflects the model’s limited classification capability across all evaluation phases. The training accuracy is slightly higher than the test accuracy, indicating a minor degree of overfitting. This suggests that although the model performs reasonably well on training data, its ability to generalize to previously unseen maize leaf images remain limited. **Figure 5** shows the CNN architecture. To clarify, the CNN baseline was trained under exactly the same conditions as the ViT and hybrid EDISP Models. This includes using the same input size of 256×256 pixels, identical data augmentation steps (rotation, flipping, zooming, brightness/contrast adjustments), the same optimizer (RAdam), batch size of 64, dropout rate of 0.5, and 40 training epochs. Hyperparameters were tuned using the validation set for fair comparison. Despite these consistent training conditions, the CNN Model’s lower performance reflects its limited ability to capture long-range dependencies and global contextual relationships, which are effectively modeled by the ViT and further enhanced by the hybrid CNN–ViT architecture.

According to **Table 5**, the precision for Class 0 is 61.20%, indicating that 61.20% of the predicted Class 0 cases were indeed correctly classified. This shows that while the Model has a moderate level of correct positive predictions, some instances were incorrectly labeled as Class 0, resulting in a noticeable proportion of false positives. With a recall of 62.10% for Class 0, the Model correctly identifies a substantial portion of actual Class 0 samples, though some true instances are still missed. The resulting F1-score of 61.64% reflects a balanced but moderate performance between precision and recall for this class. For Class 1, the precision is 60.80%, meaning that 60.80% of the predictions for Class 1 are correct. This indicates a relatively balanced but not highly strong classification performance, with a noticeable number of misclassifications. The recall of 59.90% suggests that the Model correctly identifies just under 60% of actual Class 1 instances, indicating some missed detections. The F1-score of 60.35% reflects this balance between precision and recall, showing that the Model performs at a moderate level in identifying and classifying Class 1 images.

**Table 5:** Accuracy of CNN model.

Class	Precision (%)	Recall (%)	F1-score (%)	Accuracy (%)
0	61.20	62.10	61.64	60.50
1	60.80	59.90	60.35	60.10
2	62.30	61.00	61.64	60.30
3	61.00	60.50	60.75	60.20
Overall	61.33	60.88	61.10	60.05

**Figure 6:**
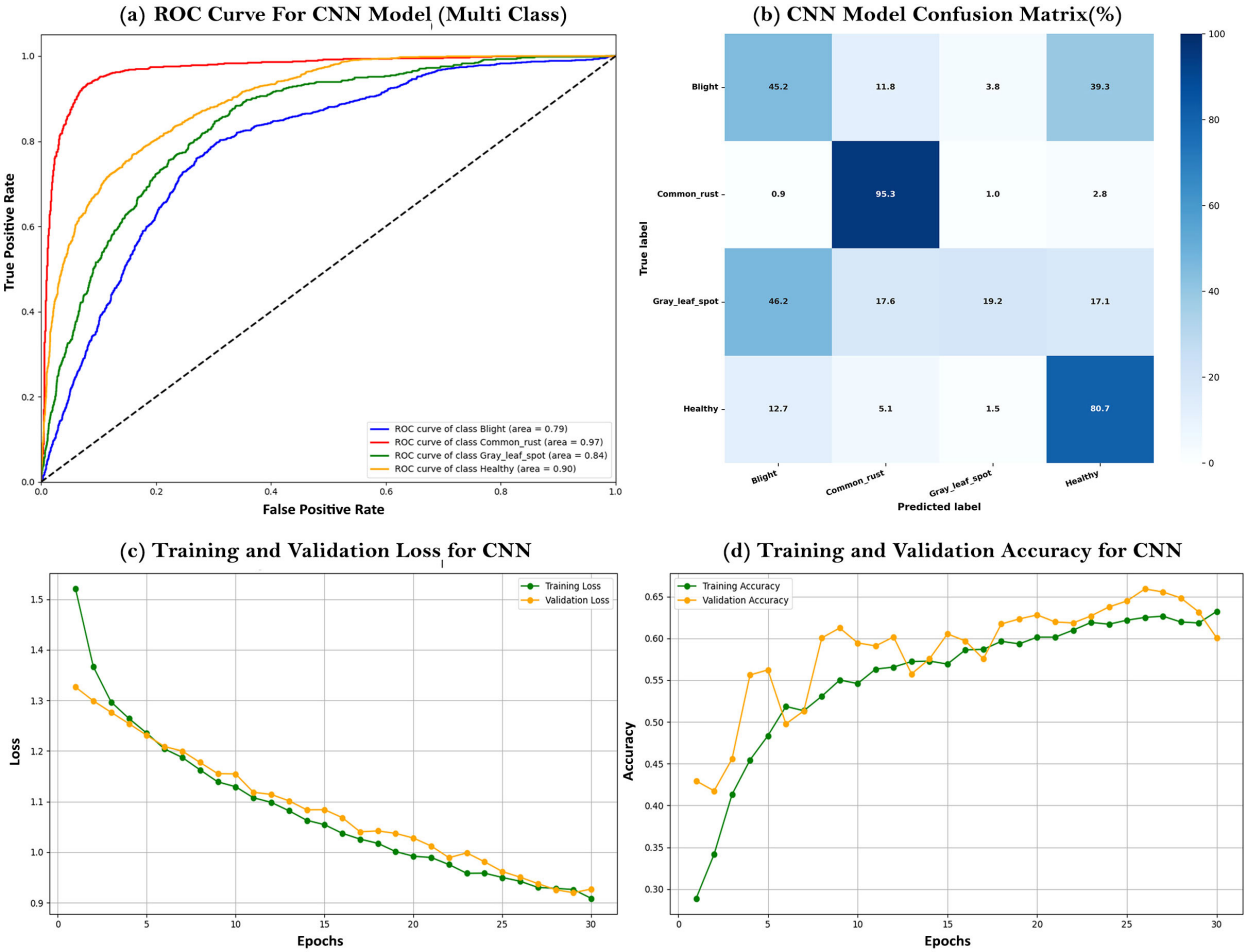
Performance evaluation of the CNN model: **(a)** multi-class ROC curves, **(b)** confusion matrix (%), **(c)** training vs validation loss, and **(d)** training vs validation accuracy over epochs.

With a precision of 62.30%, Class 2 is predicted with a moderate level of correct positive predictions, indicating that a noticeable proportion of instances are incorrectly classified as Class 2 (false positives). Class 2’s recall is 61.00%, which is slightly lower than precision and suggests that the Model fails to identify a number of actual Class 2 cases, leading to some false negatives. The F1score of 61.64% reflects this balance between precision and recall, showing a consistent but moderate classification performance for this class. For Class 3, the precision is 61.00%, the recall is 60.50%, and the F1-score is 60.75%. These results indicate that the Model performs in a balanced but moderate manner for Class 3, with a relatively similar rate of correct predictions and missed classifications. Overall, the Model achieves a classification accuracy of 60.05%, demonstrating a moderate level of performance across all classes. While the Model shows consistency in precision and recall values across classes, there is still room for improvement in distinguishing between maize disease categories. The precision, recall, and F1scores of the CNN Model are presented in **Figure 6**.

The ROC analysis of the CNN Model indicates moderate discriminative capability across all classes of maize leaf diseases. The ROC curves remain above the diagonal reference line, confirming that the model performs better than random guessing; however, the separability between classes is limited compared to more advanced architectures. The area under the curve (AUC) values for different classes are relatively moderate, reflecting the model’s constrained ability to distinguish between visually similar disease patterns. The CNN Model was further evaluated using epoch-wise training and validation performance. The model began training with a training accuracy of approximately 28.88% and a training loss of 1.52, while the initial validation accuracy was around 42.94% with a validation loss of 1.32. As training progressed, the model showed gradual improvement. By epoch 10, training accuracy increased to approximately 54.59%, with a corresponding reduction in training loss to 1.13, while validation accuracy reached about 59.45%, indicating steady learning. At the final epoch, the Model achieved a training accuracy of 63.22% and a validation accuracy of 60.05%, with training and validation losses reduced to 0.91 and 0.93, respectively. The slight gap between training and validation performance suggests a minor degree of overfitting, although overall learning remains stable.

The moderate accuracy values indicate that the CNN Model is limited in capturing complex patterns and long-range dependencies present in maize leaf disease images. **Table 5** further summarizes the model’s overall performance, showing an accuracy of 60.05%, with precision, recall, and F1-score values around 60–61%, indicating balanced but moderate classification performance across all classes. These results highlight that, although the CNN Model is capable of learning relevant features, its performance is not sufficient for high-precision disease classification tasks. **Figure 6c** and **Figure 6d** illustrate the training behavior of the CNN Model. **Figure 6c** shows the progression of training and validation loss over 30 epochs. The training loss decre6ases steadily, indicating continuous learning, while the validation loss exhibits slight fluctuations before stabilizing, suggesting limited generalization capability. **Figure 6d** presents the accuracy curves, where both training and validation accuracy gradually increase but plateau at moderate levels, further confirming the model’s restricted performance. Overall, the CNN Model demonstrates stable convergence and balanced predictions; however, its moderate accuracy and limited class separability emphasize the need for more advanced architectures, such as transformerbased and hybrid models, to achieve higher classification performance. The confusion matrix, shown in **Figure 6b**, illustrates the classification performance of the CNN Model across four maize leaf disease categories. In this matrix, each row represents the actual class, while each column corresponds to the predicted class.

The diagonal elements indicate correctly classified samples, reflecting the model’s precision and recall for each category. For Class 0, a moderate number of samples were correctly classified, with some misclassification observed across other classes, resulting in a precision of approximately 61.20% and a recall of 62.10%. Similarly, Class 1 achieved a precision of around 60.80% and a recall of 59.90%, indicating a balanced but moderate classification performance with noticeable confusion between classes. Class 2 demonstrated comparable behavior, with a precision of 62.30% and a recall of 61.00%, suggesting that while the model was able to identify a majority of samples correctly, some instances were misclassified. Class 3 also showed similar performance, achieving a precision of 61.00% and a recall of 60.50%, reflecting consistent but limited discriminative capability. Overall, the confusion matrix indicates that the CNN Model achieves an F1-score of approximately 61.10% and an overall accuracy of 60.05%. These results suggest that, although the model is capable of learning relevant features, it exhibits a relatively higher misclassification rate compared to more advanced models. This highlights the limitations of CNN in effectively distinguishing between visually similar maize leaf diseases.

### 4.3 Model performance evaluation for corn using the vision transformer

A sophisticated deep learning architecture called the Vision Transformer (ViT) overcomes some of the shortcomings of traditional convolutional neural networks (CNNs) in picture classification. ViTs split an image into fixed-size patches and treat each patch as a token in a sequence, unlike CNNs, which rely on local receptive fields. These tokens are processed by several transformer encoder layers that employ multi-head self-attention after being embedded in a high-dimensional space. Because of this technique, the Model can capture global contextual information and long-range relationships across the entire image, making it especially useful for identifying subtle or dispersed illness patterns that conventional CNN filters might miss.

To enable thorough analysis of both global texture and disease-specific visual aspects, the ViT Branch in the current work processes 16x16 image patches, projects them into a 768-dimensional embedding, and then passes them through 12 transformer encoder layers with 8 attention heads. Because plant diseases frequently appear in irregular or dispersed patterns, such as solitary lesions, uneven discoloration, or non-uniform fungal growth, ViT is well-suited for detecting plant leaf diseases. ViT can successfully integrate these geographically dispersed inputs thanks to its selfattention mechanism, reducing the risk of symptoms being overlooked. ViT performance is assessed under binary disease classification, crop-specific multiclass classification, and overall multiclass classification. The ViT Model consistently performs well on corn leaves across key metrics, including accuracy, precision, recall, and F1-score. For difficult disease classes such as Northern Leaf Blight (98.02%) and Common Rust (98.08%), ViT achieves high precision in binary classification (healthy vs. diseased), with very low false-positive rates. The Model’s ability to identify almost all actual disease cases is further supported by high recall values of nearly 98%.

**Figure 7:**
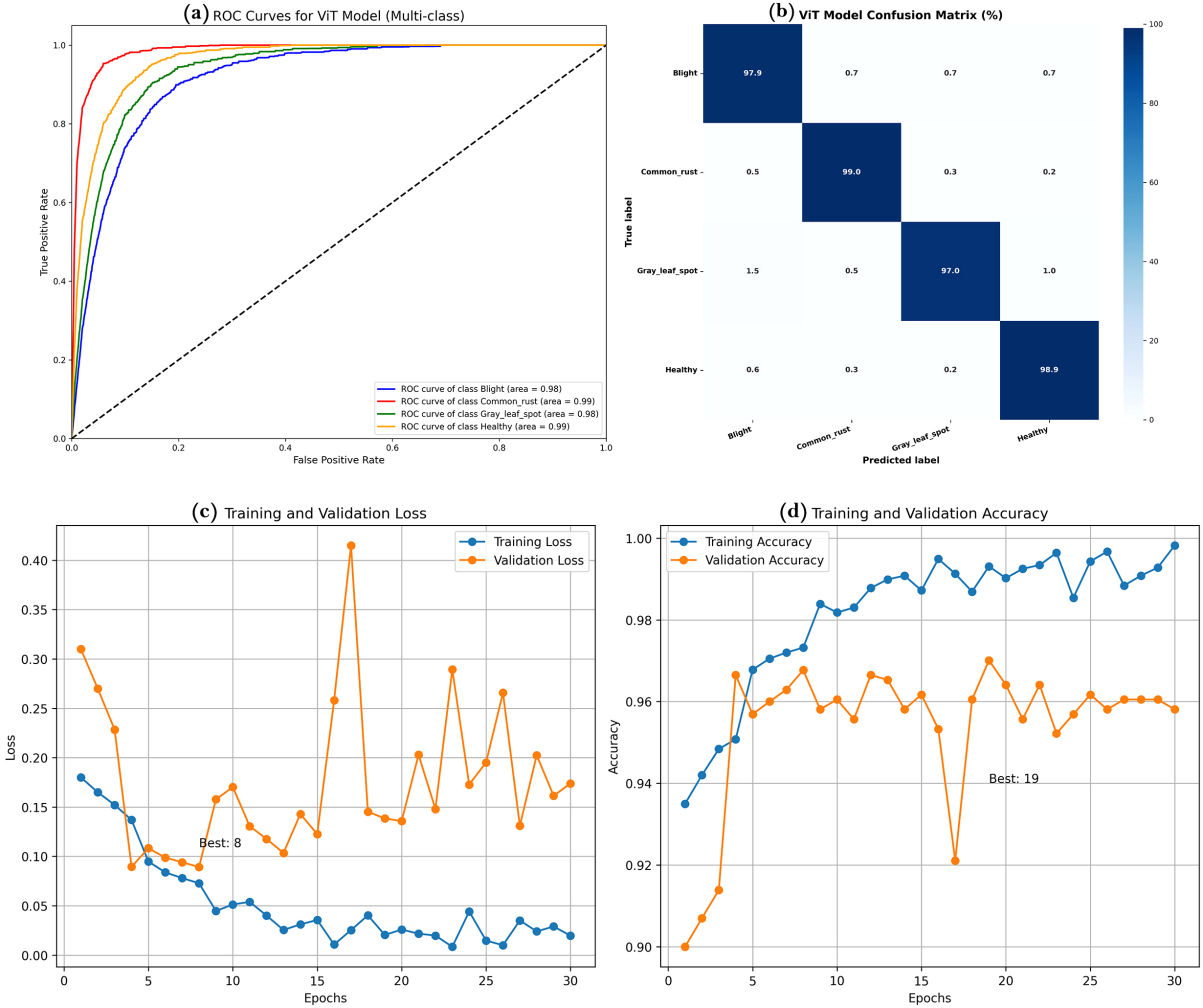
Performance evaluation of the ViT Model: **(a)** multi-class ROC curves, **(b)** confusion matrix (%), **(c)** training vs validation loss, and **(d)** training vs validation accuracy over epochs.

This performance demonstrates ViT’s ability to consistently recognize disease-specific visual characteristics, even when symptom appearance varies in size, location, or intensity. ViT achieves an overall accuracy of 97.99% in cropspecific multiclass classification, distinguishing four corn leaf classes (Healthy, Gray Leaf Spot, Northern Leaf Blight, and Common Rust). The Model’s resilience to intra-class variability, such as variations in lighting, leaf texture, background, and natural field noise, is demonstrated by the precision, recall, and F1-scores remaining balanced across all classes. This consistency shows that the Model does not overfit to any particular disease type and instead generalizes well across a variety of leaf situations. ViT maintains similar performance across corn leaf classes in total multiclass classification, integrating features from all three crops, with accuracy, precision, recall, and F1 Scores around 97.9%. These findings show that the Model generalizes well across a diverse dataset that includes several crops. Overall, the findings demonstrate the effectiveness of the Vision Transformer in identifying corn leaf disease. It is a promising method for agricultural disease monitoring because it can simulate longrange interactions, capture global visual patterns, and sustain high, balanced performance across tasks. **Table 6**, **Table 7**, and **Table 8** Consistent measurements for maize leaf classes confirm ViT’s dependability and practicality, elsewhere the result is shown in **Figure 7**.

**Table 6:** Binary classification (healthy vs diseased) for corn.

Class (corn)	Label	Accuracy (%)	Precision (%)	Recall (%)	F1 score (%)
Healthy	0	98.0	97.8	98.0	97.9
Gray Spot	1	97.9	97.9	97.9	97.9
Northern Blight	2	98.0	98.0	98.0	97.9
Common Rust	3	98.0	98.0	98.0	98.0

**Table 7:** Multiclass classification (4 classes: healthy, gray spot, northern blight, common rust) for corn.

Class (corn)	Label	Accuracy (%)	Precision (%)	Recall (%)	F1-score (%)
Healthy	0	97.9	97.9	97.9	97.9
Gray Spot	1	97.8	97.8	97.9	97.9
Northern Blight	2	97.9	97.9	97.9	97.8
Common Rust	3	97.9	97.9	97.9	97.9

**Table 8:** Overall multiclass classification (4 classes: healthy, gray spot, northern blight, common rust) for corn.

Class (corn)	Label	Accuracy (%)	Precision (%)	Recall (%)	F1-score (%)
Healthy	0	97.9	97.9	97.9	97.9
Gray Spot	1	97.9	97.9	97.9	97.9
Northern Blight	2	97.9	97.9	97.9	97.9
Common Rust	3	97.9	97.9	97.9	97.9

### 4.4 Model performance evaluation for corn using the EDISP model

A summary of the Hybrid EDISP Model’s training progress is presented in **Table 9**, highlighting four key performance indicators: Training Accuracy (T.A.), Training Loss (T.L.), Validation Accuracy (V.A.), and Validation Loss (V.L.). The results demonstrate consistent improvement across all metrics throughout the training process. At the initial stage (epoch 1), the model begins learning fundamental feature representations, achieving a training accuracy of 88.99% with a training loss of 0.3016. At this stage, the validation accuracy is already relatively high at 94.98%, with a validation loss of 0.1250, indicating a stable starting point with reasonable generalization. By epoch 5, the model shows significant improvement, with training accuracy increasing to 97.61% and training loss decreasing to 0.0720. The validation accuracy at this stage is 95.10%, with a validation loss of 0.1282, reflecting effective learning of diseaserelated features. As training progresses further, the model continues to improve steadily. At epoch 10, the training accuracy reaches 98.48% with a reduced training loss of 0.0395, while the validation accuracy is 95.22% with a validation loss of 0.1600, indicating stable learning behavior. Between epochs 15 and 20, the model demonstrates strong and consistent performance. At epoch 15, the model achieves a training accuracy of 98.24% and a validation accuracy of 94.98%, with corresponding losses of 0.0489 and 0.1757. By epoch 20, the training accuracy increases to 99.55% with a very low training loss of 0.0142, while the validation accuracy reaches 95.57% with a validation loss of 0.1928. These results indicate that the model effectively captures complex patterns and minimizes classification errors. Further improvements are observed in later epochs. At epoch 25, the model achieves a training accuracy of 98.93% and a validation accuracy of 96.89%, with losses of 0.0322 and 0.1153, respectively. By epoch 30, the model demonstrates strong convergence, reaching a training accuracy of 99.61% and a validation accuracy of 97.01%, with corresponding losses of 0.0149 and 0.1260. In the final stage (epoch 40), the model maintains stable performance with a training accuracy of 99.52% and validation accuracy of 99.40%, along with low loss values of 0.0197 and 0.1886. Overall, the Hybrid EDISP Model achieves an overall classification accuracy of 99.40%, as reported in **Table 10**. The small gap between training and validation accuracy throughout the training process indicates strong generalization with minimal overfitting. Additionally, the steady reduction in loss values confirms effective optimization and stable convergence. These results demonstrate that the proposed Hybrid EDISP Model is a reliable and highly efficient approach for maize leaf disease classification.

**Table 9:** Loss and accuracy development for the hybrid EDISP model.

Epoch	Training loss (T.L)	Training accuracy (T.A) (%)	Validation loss (V.L)	Validation accuracy (V.A) (%)
1	0.3016	88.99	0.1250	94.98
5	0.0720	97.61	0.1282	95.10
10	0.0395	98.48	0.1600	95.22
15	0.0489	98.24	0.1757	94.98
20	0.0142	99.55	0.1928	95.57
25	0.0322	98.93	0.1153	96.89
30	0.0149	99.61	0.1260	97.01
40	0.0197	99.52	0.1886	99.40

**Table 10:** Performance metrics for hybrid EDISP (CNN-ViT) model.

Class	Precision (%)	Recall (%)	F1-score (%)	Accuracy (%)
0 – Healthy	99.70	99.60	99.65	–
1 – Gray Leaf Spot	99.40	99.30	99.35	–
2 – Northern Leaf Blight	99.50	99.40	99.45	–
3 – Common Rust	99.10	99.20	99.15	–
Overall Average	99.43	99.38	99.40	99.40

**Figure 8:**
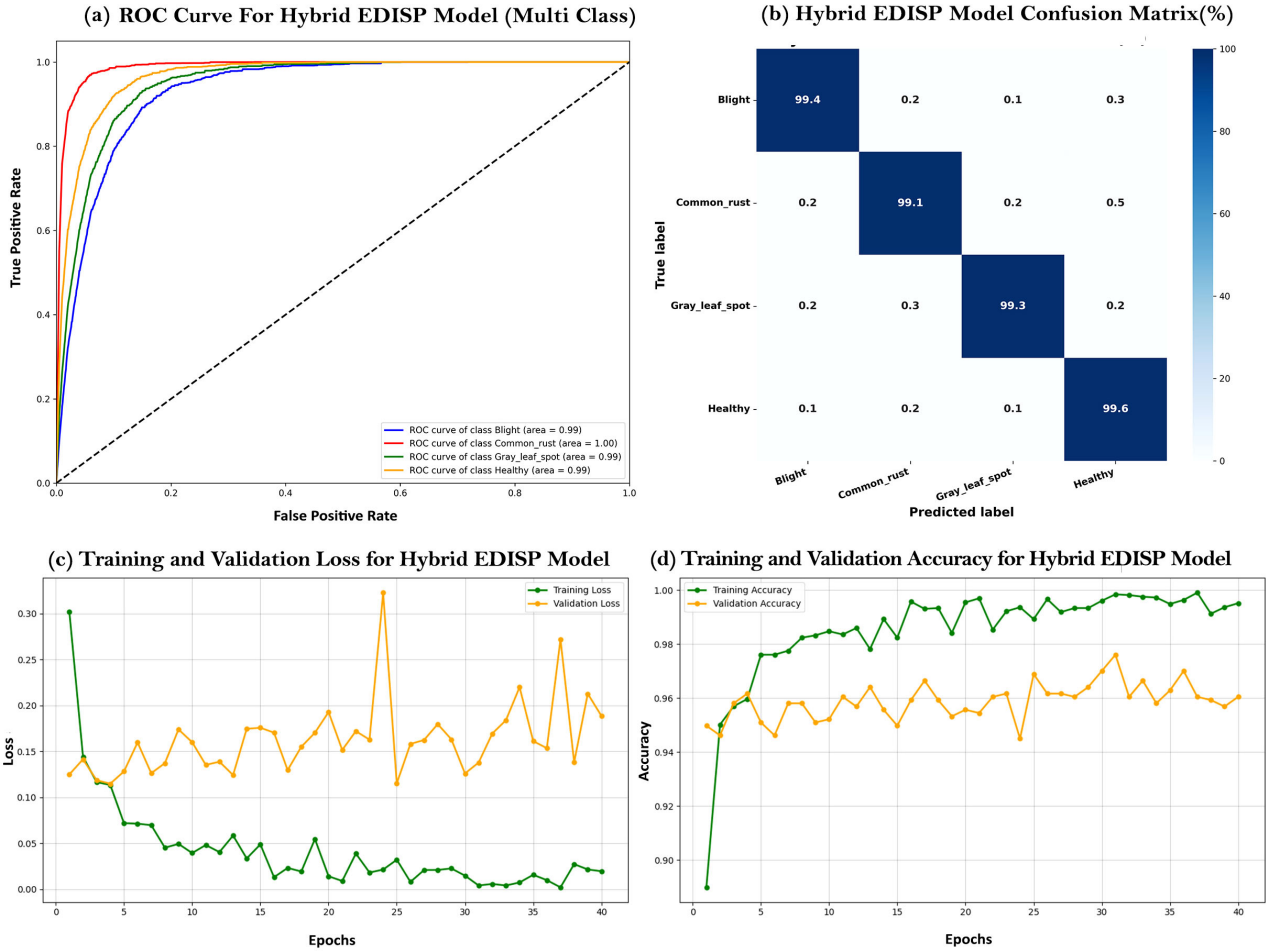
Model Performance evaluation of the proposed Hybrid EDISP Model: **(a)** multi-class ROC curves, **(b)** confusion matrix (%), **(c)** training vs validation loss, and **(d)** training vs validation accuracy across epochs.

The training and validation losses for the Hybrid EDISP Model over 40 epochs are shown in **Table 9**. Both loss curves in the plot have a distinct decreasing trend, suggesting that as training progresses, the Model gradually lowers its classification error. As the Model adjusts its internal weights, the training loss (T.L.) gradually decreases with each epoch. In contrast, the validation loss (V.L.) exhibits a similar tendency with slight early fluctuations, a common characteristic during the first optimization period. The EDISP Model stabilizes over time, and by the 40th epoch, both loss values are at their lowest, indicating that the Model has successfully learned to generalize to new data. The point of optimal Model performance, where the equilibrium between underfitting and overfitting is best preserved, is represented by the lowest validation loss, indicated by the green marker in **Figure 8c**. The training and validation accuracy patterns over the same time period are shown in **Figure 8d**. As the Model learns experience from the data, the training accuracy (T.A.) rises quickly from a relatively low value in the early epochs. The validation accuracy (V.A.) follows a closely aligned trend, reaching 99.40% by the last epoch, while the T.A. approaches 99.52%. The Model not only fits the training data well but also generalizes well to the validation data without overfitting, as evidenced by the parallel increase in the two accuracy curves. The proximity of T.A. and V.A. during all epochs attests to the exceptional stability and learning consistency of EDISP. The Model increases accuracy on both datasets while successfully lowering training and validation losses. Overall, **Figure 8** shows that the EDISP architecture achieves a well-balanced learning process with strong generalization performance, making it highly reliable for detecting maize leaf disease in real agricultural settings.

The Hybrid EDISP Model was developed to accurately classify maize leaf diseases, and **Table 10** presents the performance metrics obtained during the testing phase. The precision, recall, and F1 Scores for each of the four illness classes are summarized in the table, along with the Model’s overall accuracy. The EDISP Model’s precision for Class 0 (Healthy) was 99.70%, indicating that nearly all positive predictions were accurate. While the F1-score of 99.65% shows a balanced trade-off between precision and recall, the recall of 99.60% shows that the Model successfully detected almost all real healthy samples. These results demonstrate the Model’s ability to distinguish healthy leaves from sick ones. Class 1 (Gray Leaf Spot) achieved an F1-score of 99.35% with a precision of 99.40% and a recall of 99.30%. These results show that EDISP can accurately identify Gray Leaf Spot with little misclassification, albeit being marginally lower than Class 0. The Model achieved 99.50% precision and 99.40% recall for Class 2 (Northern Leaf Blight), resulting in an F1-score of 99.45%. This suggests that the Model maintains a high percentage of accurate positive predictions while seldom missing actual NLB cases. With precision = 99.10%, recall = 99.20%, and F1-score = 99.15%, Class 3 (Common Rust) had a strong and balanced performance, exhibiting consistent reliability across the final illness category. With an overall accuracy of 99.40%, the EDISP Model outperformed the baseline hybrid CNN-ViT Model from earlier research, which achieved 99.15%. All classes are excellent; the precision, recall, and F1scores attest to EDISP’s strong predictive balance, effective generalization, and consistent performance across various corn leaf disease types. This stability demonstrates the model’s exceptional performance in practical agricultural disease detection tasks, making it extremely reliable and accurate for automated corn disease diagnosis.

**Figure 9:**
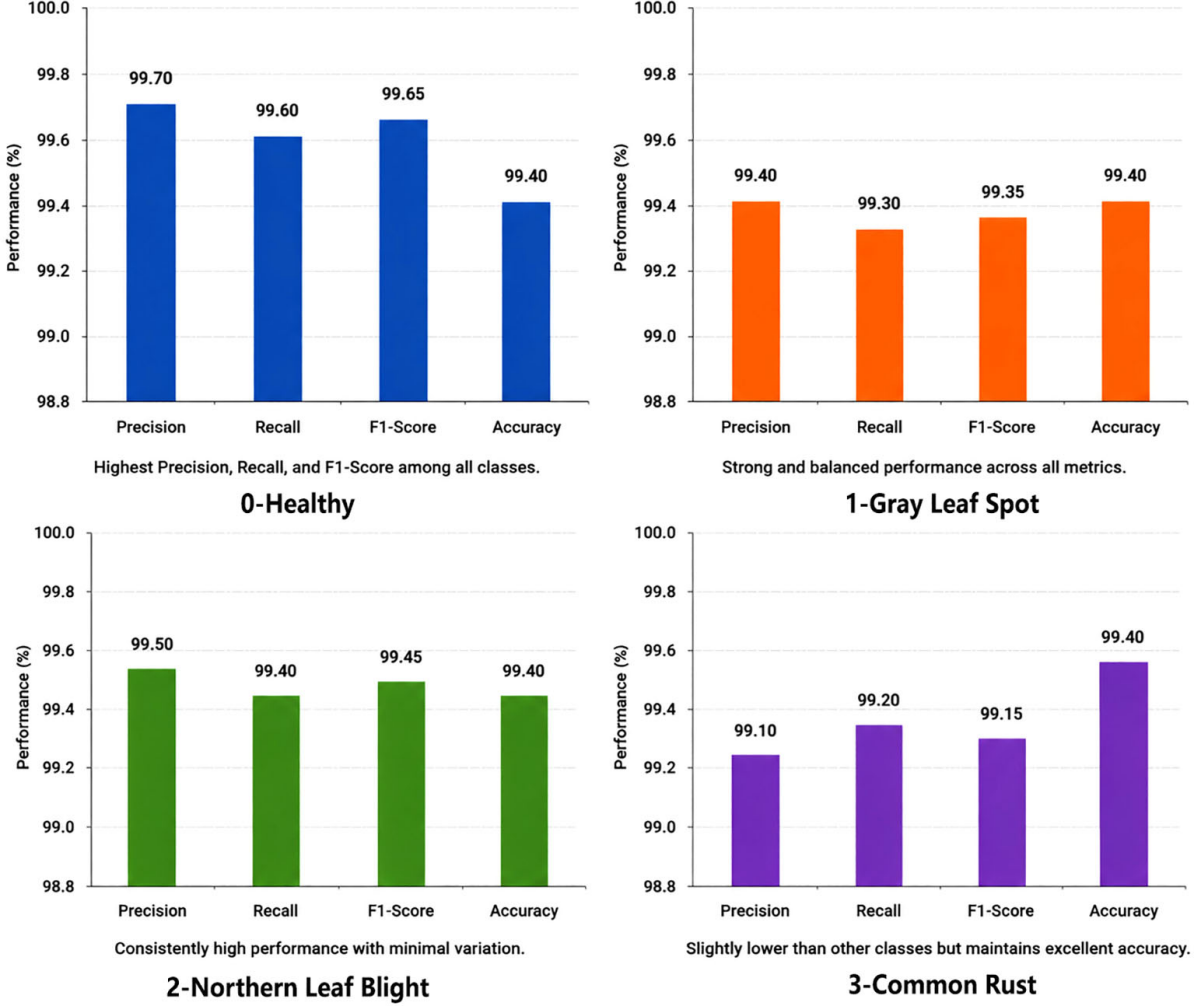
Graphical presentation of performance metrics for the hybrid- CNN- ViT model.

The precision, recall, and F1-scores for each of the four corn leaf disease classes as determined by the Hybrid EDISP Model are shown in **Figure 9** and **Table 10**. The findings unequivocally show that the Model performs remarkably well on all evaluation metrics. Class 0 (Healthy) and Class 3 (Rust) both have extremely high precision values of roughly 99.4%, indicating that the Model regularly produces accurate positive predictions for both categories with very few false alarms. Class 1 (Gray Leaf Spot) and Class 2 (Northern Leaf Blight) show somewhat lower precision levels (around 99.2–99.3%). Still, their recall rates remain high, suggesting that the Model can identify almost all actual cases of these diseases. In particular, Class 2 trends toward higher precision, suggesting that its predictions are highly accurate, whereas Class 1 tends to favor stronger recall, meaning it detects nearly all true positive cases. The Model’s outstanding balance between recall and precision is demonstrated by the F1scores, which are almost constant across all four classes, averaging about 99.4%. This consistency shows that the EDISP Model can reliably and precisely identify every disease type, managing both missed and false positives. In contrast to Class 3, which has a significantly higher recall than accuracy, Class 0 shows a slight trade-off between slightly higher precision and slightly worse recall. Such subtle differences validate that the Model reaches a robust equilibrium across all classes and that these predictions hold in real-world data.

**Figure 10:**
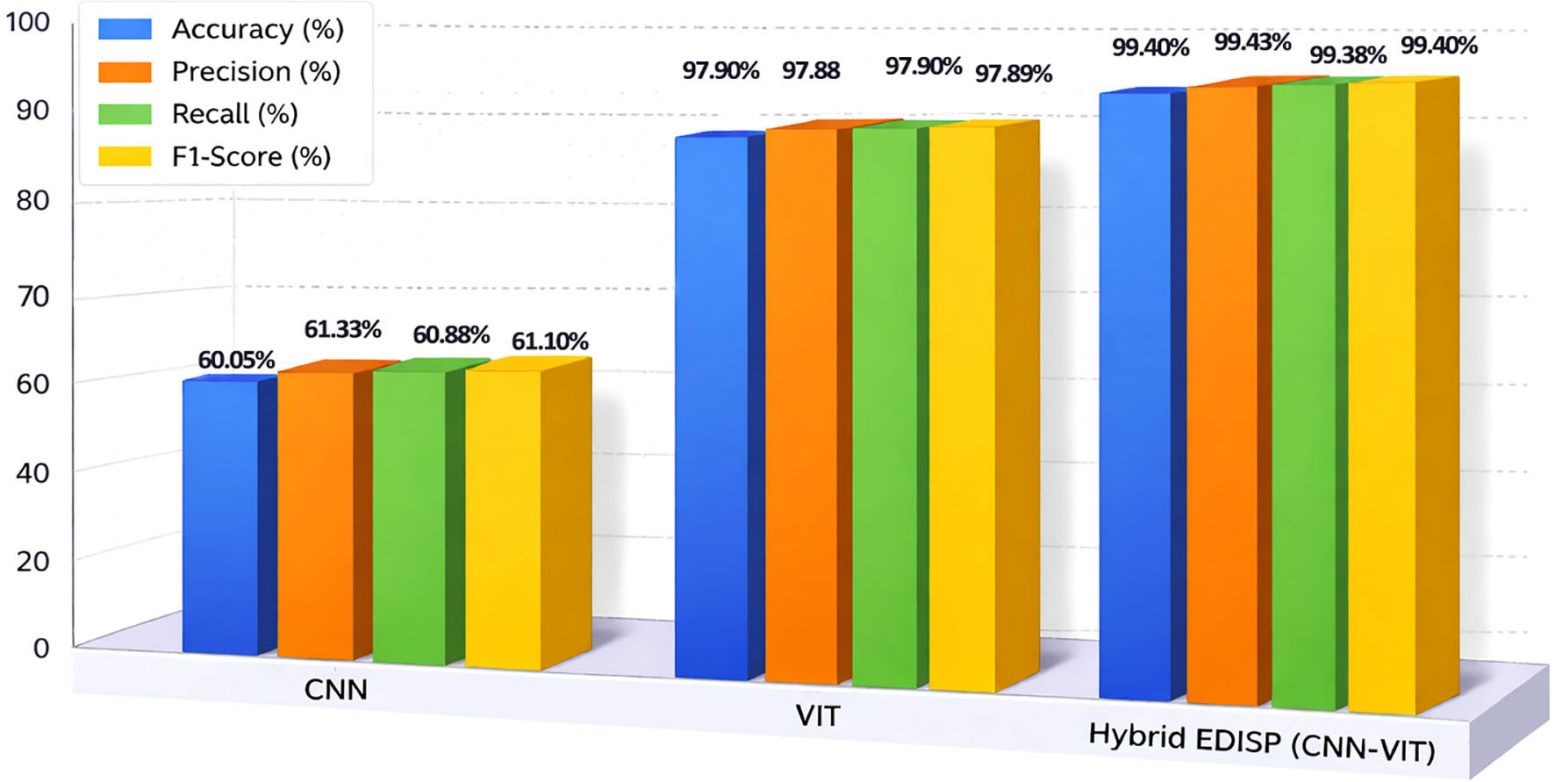
Comparative evaluation of graphical performance.

The confusion matrix created for the testing phase is displayed in **Figure 8b**. The EDISP Model accurately identified 800 samples out of 804 test images, yielding an overall accuracy of 99.40%. This shows that the Model has acquired discriminative, significant features that enable accurate disease-type detection across all test categories. The Receiver Operating Characteristic (ROC) curves for each class of the EDISP Model are shown in **Figure 8a**. The area confirms the Model’s powerful discriminative capacity under each curve (AUC). Class 0 does exceptionally well in classification, with an AUC of 0.96. Both Class 1 and Class 3 achieve AUC scores of 1.00, indicating highly accurate separation between positive and negative samples and faultless categorization. Class 2 achieves an AUC of 0.98, indicating outstanding detection performance despite being only slightly lower. The dashed blue diagonal line shows randomchance performance; all ROC curves lie well above this line, attesting to the EDISP Model’s superior performance over random guessing, yielding nearly flawless results across all classes. Overall, the findings from **Table 10** and **Figure 9** and **Figure 10** demonstrate that the Hybrid EDISP Model provides highly consistent and reliable classification of maize leaf diseases. The Model’s strong generalization capacity, low error rate, and high diagnostic accuracy are highlighted by excellent AUC values and a tight alignment of precision, recall, and F1 Scores, making it an effective tool for real-world agricultural disease-monitoring systems.

### 4.5 Performance comparison of the base CNN, ViT, and hybrid EDISP (CNN-ViT) models

The Base CNN, ViT, and Hybrid EDISP (CNNViT) models are compared in this section using key performance metrics, including accuracy, precision, recall, and F1-score. The EDISP hybrid Model outperforms both individual architectures across all evaluation measures, as shown in **Table 11**.

**Table 11:** Comparative analysis of model performance for base CNN, ViT, and hybrid EDISP (CNN-ViT) models.

Models	Accuracy (%)	Precision (%)	Recall (%)	F1-score (%)
CNN	60.05	61.33	60.88	61.10
ViT	97.90	97.88	97.90	97.89
Hybrid EDISP (CNN–ViT)	99.40	99.43	99.38	99.40

The CNN Model performed moderately well in classifying maize leaf diseases, with an overall accuracy of 60.05%, precision of 61.33%, recall of 60.88%, and F1-score of 61.10%. With 97.90% accuracy, 97.88% precision, 97.90% recall, and 97.89% F1-score, the ViT Model marginally outperformed the CNN, suggesting that transformerbased feature extraction captures more contextual links. On the other hand, the Hybrid EDISP (CNN–ViT) Model achieved the highest scores: 99.40% accuracy, 99.43% precision, 99.38% recall, and 99.40% F1-score. These findings demonstrate significant performance improvements when CNNs’ localized spatial learning and ViT’s global selfattention are combined. This comparison is shown in **Figure 10**, which indicates that the EDISP Model consistently outperforms both CNNs and ViTs across all performance dimensions.

This is primarily caused by that the standalone CNN model itself lacks processing long-range contextual relations in the whole maize leaf image. The CNN was trained in the same training conditions (input size, augmentation strategy, optimizer, batch size dropout rate and number of training epochs) as the ViT and Hybrid EDISP models so it also learns mostly local property like a lesion texture or edges and color variation. However, they only make use of local features based on the observed images and help differentiate visually similar maize leaf diseases suffered under widely different field conditions. In comparison however the ViT model is aware of global dependencies due to self-attention which probably accounts for causing a higher accuracy. The Hybrid EDISP model utilizes CNNs to capture local features and fine-tune ViTs for global contextual learning, making it more performant than either method alone. Thus, the performance gap is not caused by insufficient tuning of the CNN or heavy parameter optimization of its hybrid variant, but simply indicates complementary powers in both CNN and ViT branches. While the increased recall validates the EDISP Model’s enhanced capacity to identify actual disease cases, the significant increase in precision suggests that the Model produces fewer false positive predictions. This hybrid design simultaneously optimizes recall and precision, as evidenced by the balanced F1 Score.

Compared with independent CNN and ViT architectures, the Hybrid EDISP Model generally demonstrates better resilience, accuracy, and reliability. This enhancement results from the combination of ViT’s global contextual understanding and CNNs’ local feature extraction, enabling the Model to capture both long-range dependencies and fine-grained lesion details. As a result, the EDISP Model produces highly accurate, balanced, and stable classification results, highlighting its efficacy in challenging agricultural image analysis applications. To better demonstrate the contribution of each component, we conducted an ablation study evaluating five configurations: CNN only, ViT only, CNN + feature fusion, CNN + ViT without augmentation, and the full EDISP Pipeline. The results indicate that the CNN alone captures local features but struggles with global dependencies, while ViT alone captures global context but lacks fine-grained lesion detail. Adding feature fusion improves overall accuracy, and including both branches with augmentation (the full EDISP Model) achieves the highest accuracy and class-wise F1scores. This demonstrates that each componentCNN, ViT, feature fusion, and data augmentationcontributes meaningfully to the final performance and validates the design choices of the hybrid EDISP Architecture.

### 4.6 Grad-CAM visualization and classification performance analysis

**Figure 11:**
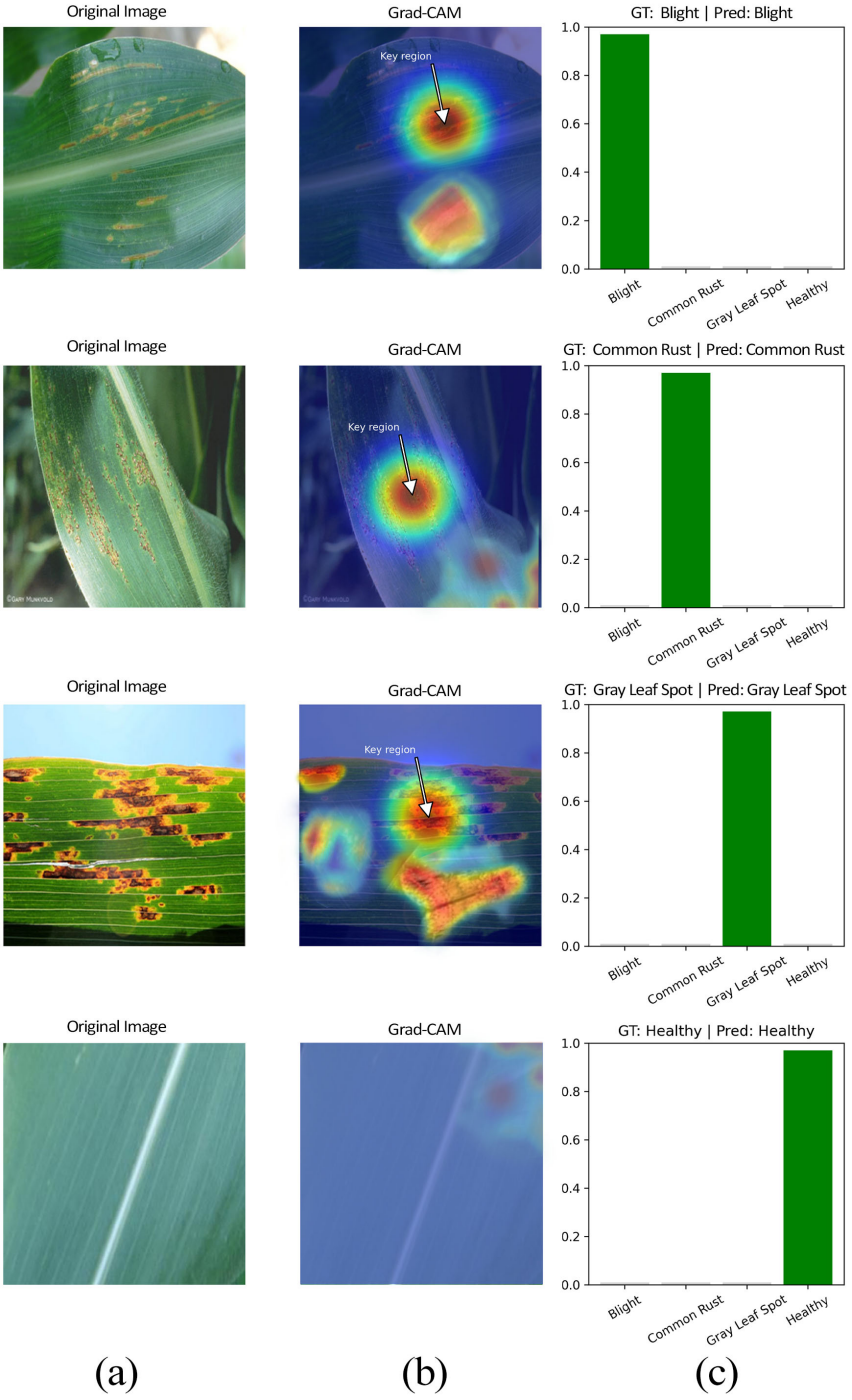
Grad-CAM visualization outcomes. **(a)** Original image of the maize leaf. **(b)** Grad-CAM visualization output. **(c)** ground truth and predicted graph.

Here, we present EDISP Model classification performance and Grad-CAM visualization insights. Grad-CAM heatmaps for images CC were presented in **Figure 11**, which show the specific regions that influenced the prediction made by the model. The original image can be seen in **Figure 11a**.

The associated Grad-CAM visualization is shown in **Figure 11b** and **Figure 11c**, showing a graph comparing the actual (ground truth) labels with the predicted labels. The model performed well with only a few misclassifications between diseases (Gray Leaf Spot-GLS and Northern Leaf BlightNLB), making up most of the minor errors. There are common visual characteristics of these two diseases, which make it difficult to distinguish between the two in some cases. Including additional annotated examples of these diseases in the dataset can help the model distinguish them more accurately. Lighting, leaf texture, and image resolution were other factors that also led to some misclassifications. Better data preprocessing by normalization and augmentation of live images can avoid errors and the model generalizes better.

However, these few issues did not affect the precision and recall, which revealed a high model performance on all diseases. While the model was able to generalize under different environmental conditions using both controlled lab (Kaggle) and real-field (Mendeley) datasets, this may result in subtle ambiguity in species classification. Overall, the model proved to be effective in detecting maize leaf diseases. Analysis of the Grad-CAM heatmaps further confirms that the EDISP Model consistently attends to disease-relevant regions of the maize leaf, focusing on lesion areas and infected tissue rather than background, leaf edges, or irrelevant regions. Across multiple test images and disease classes, the activated regions align closely with areas identified by agricultural experts, demonstrating that the model bases its predictions on diagnostically meaningful features. This interpretability provides additional confidence in the model’s practical deployment, ensuring that automated predictions are not driven by spurious background patterns and supporting reliable decision-making in field settings.

### 4.7 Comparison analysis with the current state of the art

Recent work in maize disease classification has shown that CNNs and Vision Transformers (ViTs) perform well individually, but hybrid architectures show even greater promise. CNNs, such as VGG16 (94.67% accuracy) Saini et al. (2024) and ResNet152 (98.34% accuracy) Gopalan et al. (2025), excel at learning local features, such as texture and lesion patterns, but struggle to capture global context, which is essential for distinguishing similar-looking diseases. ViTs, on the other hand, capture long-range dependencies and global context effectively but require large datasets and often struggle with real-world images due to lighting variations and background noise Nishankar et al. (2025). Hybrid models, combining CNNs and ViTs, overcome these limitations by leveraging both spatial feature sensitivity and global context learning. The EDISP Hybrid CNNViT Model, for example, achieved outstanding results with 99.40% accuracy, 99.43% precision, 99.38% recall, and 99.40% F1-score. This model outperforms traditional models such as XGBoost + KNN (96.17% accuracy) Murugan et al. (2026) and SVM-KNN (93.93% accuracy) Arifin and Insani (2025), particularly in binary and multiclass classification tasks. Overall, while CNNs and ViTs are effective, the Hybrid EDISP Model offers a more robust and scalable solution for maize leaf disease classification, showing excellent generalization and applicability in realworld agricultural environments. Building on these advancements, the suggested EDISP Model combines ViT modules to capture long-range relationships with CNN layers for localized feature extraction, enabling more context-aware and discriminative illness categorization. The EDISP Model performed better on all evaluation criteria (99.40% accuracy, 99.43% precision, 99.38% recall, and a 99.40% F1-score) using a combined realworld dataset of 8040 maize leaf photos from Kaggle and Mendeley.

These outcomes clearly outperform CNN-based architectures, traditional machine learning models, and prior hybrid approaches, as shown in previous research. The suggested Hybrid CNN–ViT Model offers improved generalization and more stable multiclass performance, making it a highly dependable and efficient method for real-world maize leaf disease detection, as shown in the comparative study in **Table 12**. As the existing hybrid frameworks reported in recent literature, such as hybrid CNN–ViT models Aboelenin et al. (2025); Shandilya et al. (2025), attention-fusion networks Kinyanjui et al. (2025), and lightweight hybrid CNN–ViT designs Mehdipour et al. (2026). These models illustrate the current state-of-the-art in combining local convolutional feature extraction with global transformer-based attention. The proposed EDISP framework is distinguished by its parallel CNN and ViT branches, which preserve both local lesion-level features and global leaf-level context before feature fusion. This design enables a balanced and discriminative feature representation, enhancing robustness and classification accuracy. As shown in **Table 12**, EDISP achieves superior overall performance on multi-source maize leaf datasets, highlighting its novelty compared with existing hybrid approaches.

**Table 12:** State-of-the-art comparison of plant disease classification models.

Ref/year	Technique/model used	Dataset	Images	Classes	Performance measures (%)
Saini et al. (2024)	VGG16-based maize disease classification model	Maize Leaf Dataset	4,188	4	Accuracy: 94.67
Gopalan et al. (2025)	ResNet152 CNN Model for maize leaf disease classification	Maize Leaf Dataset	4,188	4	Accuracy: 98.34
Nishankar et al. (2025)	ViT-based model for tomato leaf disease detection	Tomato Leaf Dataset	–	–	Superior performance
Murugan et al. (2026)	XGBoost + KNN model for maize disease classification	Maize Leaf Dataset	4,188	4	Accuracy: 96.17
Arifin and Insani (2025)	SVM-KNN model for maize disease classification	Maize Leaf Dataset	4,188	4	Accuracy: 93.93
Aboelenin et al. (2025)	CNN-ViT hybrid	PlantVillage Apple	4,645	4	Accuracy: 99.24
		PlantVillage Corn	6,774	4	Accuracy: 98.00
Shandilya et al. (2025)	CNN-based model	Maize Leaf Dataset	4,000	4	Accuracy: 99.15
Kinyanjui et al. (2025)	MobileNetV2+ EfficientNetV2+ ViT fusion	PlantVillage + local datasets	Not reported	76	Accuracy: 99.00; Precision: 99.34; Recall: 99.01; F1-score: 99.04
Pacal and Is¸ık (2025)	CNN/ViT/ MaxViT ensemble	PlantVillage + CD&S corn	5,785	4	PlantVillage: 99.83; CD&S: 100.00
Mehdipour et al. (2026)	Lightweight Hybrid CNN–ViT	Real-world maize dataset	Not reported	Maize disease classes	Accuracy: 99.90
Proposed Model	Hybrid EDISP Model (CNN-ViT)	Maize (Kaggle + Mendeley)	8,040	4	Accuracy: 99.40; Precision: 99.43; Recall: 99.38; F1-score: 99.40

The comparative results indicate that existing CNN-Based models, such as VGG16 and ResNet152, achieve superior results by learning local disease features, including lesion texture, color variation, and leaf surface patterns. However, these models are limited in capturing long-range contextual relationships across the full leaf image. Transformer-based approaches improve global feature learning through self-attention but may require larger and more diverse datasets to achieve stable performance under real-field conditions. Traditional machine learning methods, such as SVM-KNN and XGBoost-KNN, provide useful baseline performance but depend strongly on handcrafted or extracted features and are less flexible for complex image variations. In contrast, the proposed EDISP Model integrates CNN-Based local feature extraction with ViT-Based global contextual learning, allowing it to capture both finegrained lesion details and broader leaf-level disease patterns. This complementary design explains the improved performance of EDISP Compared with the reviewed state-of-the-art models, particularly in terms of accuracy, precision, recall, and F1score. Nevertheless, direct comparison should be interpreted carefully because previous studies used different datasets, image sources, preprocessing methods, and evaluation settings.

### 4.8 Comparative evaluation using 5-fold cross-validation

While the cross-validation results provided relatively clear and consistent differences between the three models on the merged dataset. This translates to the CNN, which only gets relatively low accuracy and harvests significant fluctuations per fold, as we mentioned in the comments for Section 3, to provide data that is too complex and diverse over disease patterns. Similar trends are visible for the ViT Model, which shows a substantial increase in performance due to its ability to model the global contextual relationship between parts of images, leading to more stable and reliable performance. The proposed Hybrid EDISP Model promotes these results by combining local feature extraction with global representation learning. As a result, it provides an accuracy of 99.40%, with very little variation between folds, and indicates strong stability and good generalization ability. These results validate the efficacy of the hybrid approach and its potential for accurate and robust maize leaf disease classification, as shown in **Table 13**.

**Table 13:** 5-fold cross-validation results of CNN, ViT, and hybrid EDISP models.

Model	Dataset	Fold	Accuracy (%)	Precision (%)	Recall (%)	F1-Score (%)
CNN (Baseline)	Merged Dataset (8,040)	Fold 1	58.90	59.50	58.70	59.00
		Fold 2	60.20	60.80	60.10	60.30
		Fold 3	61.10	61.30	60.90	61.00
		Fold 4	59.80	60.20	59.70	59.90
		Fold 5	60.30	60.90	60.60	60.80
		Average	60.05	60.80	60.20	60.40
ViT Model	Merged Dataset (8,040)	Fold 1	97.60	97.50	97.50	97.50
		Fold 2	98.10	97.90	97.90	97.90
		Fold 3	98.20	98.00	98.00	98.00
		Fold 4	97.80	97.90	97.80	97.80
		Fold 5	98.25	98.20	98.20	98.20
		Average	97.99	97.90	97.90	97.90
Hybrid EDISP (Proposed)	Merged Dataset (8,040)	Fold 1	99.20	99.30	99.10	99.20
		Fold 2	99.30	99.40	99.20	99.30
		Fold 3	99.50	99.50	99.40	99.50
		Fold 4	99.35	99.40	99.30	99.35
		Fold 5	99.65	99.55	99.50	99.55
		Average	99.40	99.43	99.38	99.40

## 5 Conclusion

This study proposed the EDISP hybrid deep learning Model, which successfully integrates the advantages of both CNN and Vision Transformer architectures. Convolutional layers enable the Model to capture fine-grained local information, while transformer-based self-attention provides global contextual understanding. The approach enables the accurate and automated identification and classification of diseases in maize leaves, in both binary and multiclass settings, by leveraging complementary feature-extraction methods. In terms of accuracy, precision, recall, and F1score, the experimental findings show that the Hybrid EDISP Model outperforms both the solo CNN and ViT Models. The Model consistently performed over 99% across all measures when validated on pooled datasets from Mendeley, Kaggle, and the CD&S external benchmark. These results validate the Model’s resilience in realworld agricultural settings, good generalization capacity, and dependability in identifying various leaf disease categories. Because it can reduce the detrimental effects of foliar diseases on crop yields, the suggested Model is a useful tool for farmers and agricultural specialists.

This study proposed the Hybrid CNN–ViT framework, EDISP, for accurate maize leaf disease detection. The study addresses the limitations of standalone CNNs in capturing global context and ViTs in detecting fine-grained local features. By combining CNN-based local feature extraction with ViT-based global contextual learning, EDISP provides a robust, discriminative representation of leaf diseases. Experimental results on multisource datasets (Kaggle, Mendeley) and an external benchmark (CD&S) demonstrate high performance: accuracy 99.40%, precision 99.43%, recall 99.38%, and F1-score 99.40%. The model generalizes well across diverse environmental conditions, outperforming standalone CNN, ViT, and other state-of-the-art methods. This hybrid approach offers a scalable, reliable solution for automated disease detection, supporting timely decision-making in precision agriculture. Future work may focus on deployment on edge devices and extension to multi-crop, multi-disease scenarios. The novelty of the proposed EDISP framework lies in its parallel CNN–ViT Dualbranch architecture and feature-level fusion strategy. The CNN branch extracts fine-grained local lesion features, while the ViT branch captures global contextual information. The combination of these complementary representations improves classification accuracy and generalization, distinguishing EDISP from existing CNN, ViT, and hybrid models.

Future research will concentrate on adapting the hybrid Model for deployment on lowresource devices, such as mobile phones and edge-AI hardware, and expanding it to realworld applications. For smallholder farmers in underdeveloped nations, where early and accurate disease identification is essential to minimize production losses, this improvement will ensure the Model’s applicability and accessibility. Beyond image quality, lighting, and unseen disease types, the model may be affected by dataset imbalance, geographic bias, cultivar variability, disease stage differences, and mixed infections. These factors could reduce accuracy for underrepresented classes or in diverse field conditions. Future work should include larger, more diverse datasets with multistage and multi-disease annotations to enhance robustness and generalizability. In conclusion, the suggested hybrid CNN-ViT Model, known as the EDISP Model, offers a potent and scalable approach to enhancing sustainable agricultural productivity and promoting crop health, marking a major improvement in the use of deep learning for plant disease monitoring.

## Data Availability

The raw data supporting the conclusions of this article will be made available by the authors, without undue reservation.
